# Diversity of vegetable landraces in the Pampa biome of Brazil and Uruguay: utilization and conservation strategies

**DOI:** 10.3389/fpls.2023.1232589

**Published:** 2023-11-10

**Authors:** Mercedes Rivas, Rafael Vidal, Raquel Silviana Neitzke, Daniela Priori, Natália Almeida, Irajá Ferreira Antunes, Guillermo A. Galván, Rosa Lia Barbieri

**Affiliations:** ^1^Departamento de Biología Vegetal, Facultad de Agronomía, Universidad de la República., Montevideo, Uruguay; ^2^Centro Universitario Regional del Este (CURE), Universidad de la República., Rocha, Uruguay; ^3^Instituto Federal de Educação, Ciência e Tecnologia Sul-rio-grandense (IFSul), Bagé, RS, Brazil; ^4^Centro de Ensino Superior Riograndense (CESURG), Beira Campo, Sarandi, RS, Brazil; ^5^Departamento de Sostenibilidad Ambiental, Instituto Tecnológico Centro Sur, Universidad Tecnológica del Uruguay (UTEC), Durazno, Uruguay; ^6^Embrapa Clima Temperado, Pelotas, RS, Brazil; ^7^Departamento de Producción Vegetal, Centro Regional Sur (CRS), Facultad de Agronomía, Universidad de la República, Progreso, Canelones, Uruguay

**Keywords:** agrobiodiversity, agroecology, custodian farmers, landraces, Pampa biome, plant genetic resources, sustainability

## Abstract

The historical meeting between originally American people, European colonizers, and slaved peoples from Africa in the biome Pampa in South Brazil and Uruguay involved a cultural syncretism and a great genetic diversity of landraces of cultivated species. Genetic richness evolved by selection and cultural processes in response to different environments, cultural backgrounds, and needs. This review summarized the knowledge on vegetable and maize landraces in South Brazilian and Uruguayan Pampa biome, to design a strategy towards the rediscovery, conservation, and sustainable use. Landraces diversity maintained *in situ* and *ex situ* is described, specific case studies are presented, and the main problems and tools towards landraces re-valorization are discussed. We show that traditional family farming systems maintain diverse vegetable species, mainly squashes (*Cucurbita* spp.), sweet potato (*Ipomoea batatas*), beans (*Phaseolus* spp.), onion (*Allium cepa*), peppers (*Capsicum* spp.), tomato (*Solanum lycopersicum*), next to leafy vegetables and maize, among others. We propose the priority of systematic surveys as a basis for monitoring genetic erosion, increasing complementariness between *in situ* and *ex situ* conservation, and implementing sustainable conservation and utilization. The case studies highlight genetic diversity within each cultivated species, with different crop physiological responses; disease resistances, different quality traits and associated uses, from animal feeding in maize to a range of culinary uses linked to traditional culture in maize and vegetable species, and ornamental uses of specific *Capsicum* and *Cucurbita*. Some landraces were the basis for breeding, where improved cultivars allowed the competitiveness of landrace germplasm in the markets. Renewed industrialized products allowed the competitive and sustainable use of *Capsicum* landraces in Brazilian farmers communities. Strategies towards *in situ* conservation improvements and valorization are discussed; emphasizing the role played by agroecology, community seed banks and custodian farmers, participatory plant breeding, promotion of landraces specialties among consumers, and the need of research and capacity building, among others. Farmers’ participation in the decisions is a key factor, along with the academia and the public sector. Landraces and associated knowledge are treasures to be used to benefit from farmers to consumers, directing the course of agriculture towards sustainable directions.

## 1 Introduction

The Pampa biome, also known as the Río de la Plata Grasslands region (Soriano, 1991), comprises the Uruguayan territory, the southern part of the state of Rio Grande do Sul in Brazil and central-eastern Argentina (Allen et al., 2011; Mengue et al., 2020). It is recognized worldwide for its diversity of grasslands ecosystems and high diversity of C3 and C4 grass species (Boldrini, 2009). Livestock farming is the main activity in this region, which currently has 16 million hectares of *campos*, while industrial agriculture and forestry cover 11.5 and 2 million hectares, respectively (Baeza et al., 2022). In this context, the cultivation of horticultural crops is secondary in land use and is not a relevant exporting agricultural sector. However, it is relevant for self-consumption and to supply local markets and cities, together with other productive activities and with varying levels of professionalization and business development.

From the very origins in various cultures, agriculture was based exclusively on the currently so-called landraces, until formal breeding was implemented in the 20th century (Frankel and Bennett, 1970; Harlan, 1975; FAO, 1998). These landraces, understood as dynamic populations of cultivated species maintained by farmers over prolonged periods of time, are genetically diverse populations locally adapted. Landraces have productive stability, lower input requirements, and are associated with traditional management practices and uses by farmers. These characteristics together make landraces a valuable component of agrobiodiversity (Zeven, 2000; Camacho-Villa et al., 2005). It is in traditional family agroecosystems where landraces are mostly conserved, integrating the rural and biocultural landscapes of this study region. This logic consequently leads to the evolution and conservation of landraces and has resulted in a great genetic diversity that is currently conserved in the hands of farmers around the world (Jarvis et al., 2008).

In the Brazilian-Uruguayan Pampa, horticultural landraces as a result of the encounter between indigenous peoples, Europeans and Africans, many plants were adapted to cultivation in this environment, in a process of cultural syncretism, which gave rise in the Pampa biome to landraces of several cultivated species (Barbieri et al., 2014; Rivas and Condón, 2015). These landraces are a repository of valuable genes for both family farming and breeding, as they are still evolving and could become a key factor in the response to global climate change and new cultivars for modern and market-oriented vegetable production systems (Mercer and Perales, 2010; Tapia et al., 2015).

The presence of archaeological findings of maize, cucurbits and beans, and in some cases peanuts, dated as early as 4900 years BP (Iriarte et al., 2001; Iriarte et al., 2004; López Mazz et al., 2014; del Puerto et al., 2016a; del Puerto et al., 2016b) are linked to the transhumant movements of indigenous peoples in prehistoric times and particularly to the Tupi-Guarani expansion that reached the Río de la Plata (BroChado, 1984; González and Rodríguez Varese, 1990; Iriarte et al., 2017). They carried the cultivation of cassava, beans, sweet potatoes, maize and squashes. Although none of these species are native to the Pampa, the Guarani peoples brought them from elsewhere, as they occupied a much larger territory, moving in Brazil from the current state of São Paulo to Rio Grande do Sul, passing through Uruguay, Argentina, Paraguay and Bolivia, maintaining contact with other ethnic groups that occupied large areas in the Americas (Mazurana et al., 2016).

This process continued with the Spanish-Portuguese conquest that began in the 16th century. After the Jesuitic missions ended in the 18th and 19th centuries, a great migratory flow of indigenous peoples (mainly Guaraní) took place towards the territory of the *campos* (González and Rodríguez Varese, 1990; Pellegrino, 2013/2014). With the arrival of the missionary Indians their seeds came and their knowledge on agricultural practices as well as of the native flora was of great importance for the incipient development of the colonial society. The agriculture carried out in small areas or gardens supplied the incipient urban centers with maize, wheat, and some legumes (González and Rodríguez Varese, 1990). At the end of the 18th century, Félix de Azara – a Spanish military officer, engineer, and natural scientist - reported that ‘their main resource was the cultivation of maize, beans, pumpkins, peanuts, sweet potatoes and cassava’.

From the 16th century onwards, and in several waves until the 20th century, there was an influx of people from different ethnic groups and cultures of the Old World, generating a cultural mixture that also involved crops and landraces. Migration trends led to the movement of 56 million people out of the European continent between 1821 and 1932, most of them to the Americas. Of the 12 million people whose destination was Latin America, 42% went to the Brazilian - Uruguayan Pampa (Pellegrino, 2013/2014). For the Pampa biome in Brazil, the main groups of European immigrants were Portuguese, Italians, Germans, followed by Pomeranians and Spanish (Barbieri et al., 2014); while for Uruguay were Spanish and Italians, followed by people from several nationalities, like French, Basque, English, Swiss, Syrian Lebanese, Slavs, Armenians, and Russians (Vidart and Pi Ugarte, 1969).

Africans, brought into the region as slaves, also played a role in this cultural intermingling. It is estimated that some 70,000 people arrived in the Río de la Plata between 1777 and 1812. In the *campos*, the slaves were not exclusively dedicated to livestock farming, but also carried out small-scale agriculture (Borucki et al., 2012). Once slavery was abolished or after having escaped, Afro-descendants went to live in settlements called *quilombolas*, in the south of present-day Brazil, or in the *rancheríos* in Uruguay, while other African descendants stayed as labors on cattle ranches. Agriculture, especially for family consumption, is a common practice in these communities, mainly due to their strong cultural bond with the land. The cultivation of edible species, especially landraces of squashes, sweet potatoes, cassava, maize, and beans, is prioritized for food security, through strategies learned from their ancestors (Mazurana et al., 2016; Febrero and Marín, 2022).

In short, vegetable landraces in this region evolved and were maintained based on the dynamics of traditional family production and syncretism between different cultures until the second half of the 20th century. From the 1970s and the 1980s a period marked by changes in production systems began, characterized by a continuous process of rural-to-urban migration that persists to these days, causing the aging of the rural population and the abandonment/withdrawal of thousands of family farms. These demographic processes are accompanied by the replacement of landraces by a reduced group of commercial cultivars with higher input requirements (Comité Nacional de Recursos Fitogenéticos, 2021; Abreu et al., 2022). These processes of genetic erosion are associated with losses of cultural diversity such as local traditional knowledge about cultivation practices, seed selection, forms of consumption and other uses, traditional dishes and festivals linked to some of these crops and landraces. The loss of agrobiodiversity affects the adaptive capacity and evolutionary potential of crops, as well as the resilience and functionality of agroecosystems, causing vulnerability to adverse factors like climate change, food insecurity, and ultimately affecting the lives of farmers and rural communities (Sthapit and Padulosi, 2012; Azeez et al., 2018; Khoury et al., 2022). This genetic erosion refers to the loss of within species genetic diversity and to the abandonment of some crops (NUS, Neglected and Underutilized Species) (Padulosi et al., 2012; Barbieri et al., 2014; Hernández et al., 2019).

Genetic and agronomic information on landraces is globally scarce, thereby limiting their utilization, management, and conservation (Azeez et al., 2018). This situation is not dissimilar to the challenges faced in the Pampa biome. To address this, it is crucial to establish an inventory of landraces conserved *in situ* and *ex situ*, gather information on the degree of genetic erosion, explore forms of utilization and valorization, and incorporate local knowledge and legal and promotional mechanisms that can support their protection and promotion (Khoury et al., 2022).

The aim of this work is to contribute to a diagnosis of the state of landrace plant genetic resources in the Pampa biome region of Brazil and Uruguay, which would allow us to contribute to the design of a strategy for their conservation and sustainable use. Particularly, (i) the knowledge about *in situ* and *ex situ* conservation of landraces was gathered and analyzed, as well as their utilization and the ways of valorization, and (ii) the identified main limiting constraints to set the landraces in value and resignification were described, in a context of agroecology and farmers’ right consideration.

## 2 Methodology

**Figure 1:**
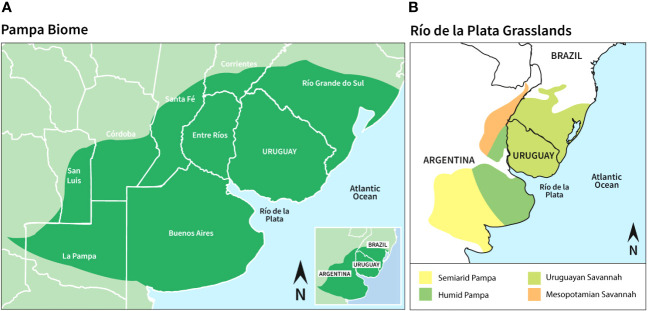
**(A)** Geographical location of the Pampa biome, **(B)** Classification of the Río de la Plata Grasslands (Design: Laura Moraiti).

The area of study comprises the territory of *Pampa* biome in South Brazil and Uruguay (**Figure 1**). Based on geomorphological, edaphic and plant community characteristics, the region of the northern and southern *campos* is grouped under the name of the Uruguayan Savannah or Uruguayan district of the Pampean province (Chebataroff, 1951; Cabrera and Willink, 1973). The *campos* are characterized by grasses, herbs, small shrubs and occasional trees in an undulating, hilly landscape (Overbeck et al., 2007; Allen et al., 2011). The temperate-subtropical climate is humid, warm in summer and mild in winter, mostly of the Cfa type (Peel et al., 2007).

The methodology employed in this study was a scoping literature review (Munn et al., 2018) to synthesize and analyze indexed publications and gather gray literature (theses, reports, promotion and exchange networks) on vegetable landraces in Uruguay and the southern region of the state of Rio Grande do Sul (Brazil).

The review focuses on (1) characterization of the productive agricultural systems where landraces are conserved *in situ* (acreage, combination of activities), farmers profile (age, gender, family history, reasons to keep their own seeds, among other issues), vegetable crops maintained as landraces (analyzing the number of species and landraces and their diversity among territories); (2) assessing the state of *ex situ* conservation; (3) presenting crop case studies in which more in-depth research has been conducted; and (4) exploring the different approaches to conserving and valorizing local vegetable genetic resources that have been implemented, including farmers seeds networks and governmental regulations.

To describe the state of *in situ* conservation, literature referred to specific vegetable species were analyzed, as well as others with an integral approach on the agrobiodiversity at a territorial level. To compare the agrobiodiversity of three surveys in Uruguayan areas where a similar methodology was followed to assess *in situ* landraces conservation, the Shannon-Wiener diversity index (H′) based on the frequency of species occurrence was estimated for each surveyed area; evenness was calculated as E = H′/lnS, where S represents the total species richness (Magurran, 1988; Magurran and McGill, 2011); and beta index was calculated as the Sørensen similarity index. A correspondence analysis was applied to analyze the association between vegetable crops with the three surveyed territories. Farmers organizations and networks that conserve landraces *in situ* were consulted to find out or check the information. We also seek information on the productive systems, socio-economic characteristics of the farmers and their families, the diversity of landraces, uses and associated knowledge.

The state of *ex situ* conservation of landraces was based on information given by genebanks, both national and institutional collections. Information on community seed banks or *casas de semillas* (seed-houses) is also presented, and the interaction between *ex situ* and *in situ* conservation is discussed.

Throughout four crop-based case studies (onion, maize, Cucurbits and *Capsicum* peppers), the general context of landraces cultivation is analyzed in a deeper manner. These cases were defined in attention to the knowledge accumulated by our research teams. Landrace phenotypic and genetic diversity, farmers’ uses and knowledge, and the state of conservation are presented and discussed, as well as information on nutritional quality, resistance to diseases, culinary uses, among other traits.

To analyze the state of the art and develop proposals for a strategy and an action plan to conserve and valorize vegetable landraces, the authors engaged in exchanges and collaborative workshops involving public institutions, civil society, and farmers organizations. Among these workshops, those organized in the frame of Latin American Network for the implementation of the International Treaty (LANIIT) project stand out, carried out by a team from Brazil, Paraguay and Uruguay (2015-2019). In addition, we highlight seminars organized by Facultad de Agronomía in Uruguay; those organized annually by Embrapa Clima Temperado in Pelotas, Brazil; workshops organized by the ‘Red Nacional de Semillas Nativas y Criollas’, and other more specific meetings in the frame of the studies on maize races and landraces in the lowlands of South America. Besides, the authors participated in the country reports on the situation of plant genetic resources for food and agriculture, presented to the ITPGRFA (International Treaty on Plant Genetic Resources for Food and Agriculture), which allowed contact and exchange networks, facilitating the information and identifying information gaps.

Regarding the situation and state of knowledge on landraces in the Brazilian - Uruguayan Pampa, research priorities were proposed to establish a baseline, whereas appropriate strategies for valorizing and conserving the agrobiodiversity of vegetable landraces were discussed.

## 3 Diversity, conservation, and utilization of vegetable landraces in the bioma Pampa

### 3.1 *In situ* conservation

**Figure 2:**
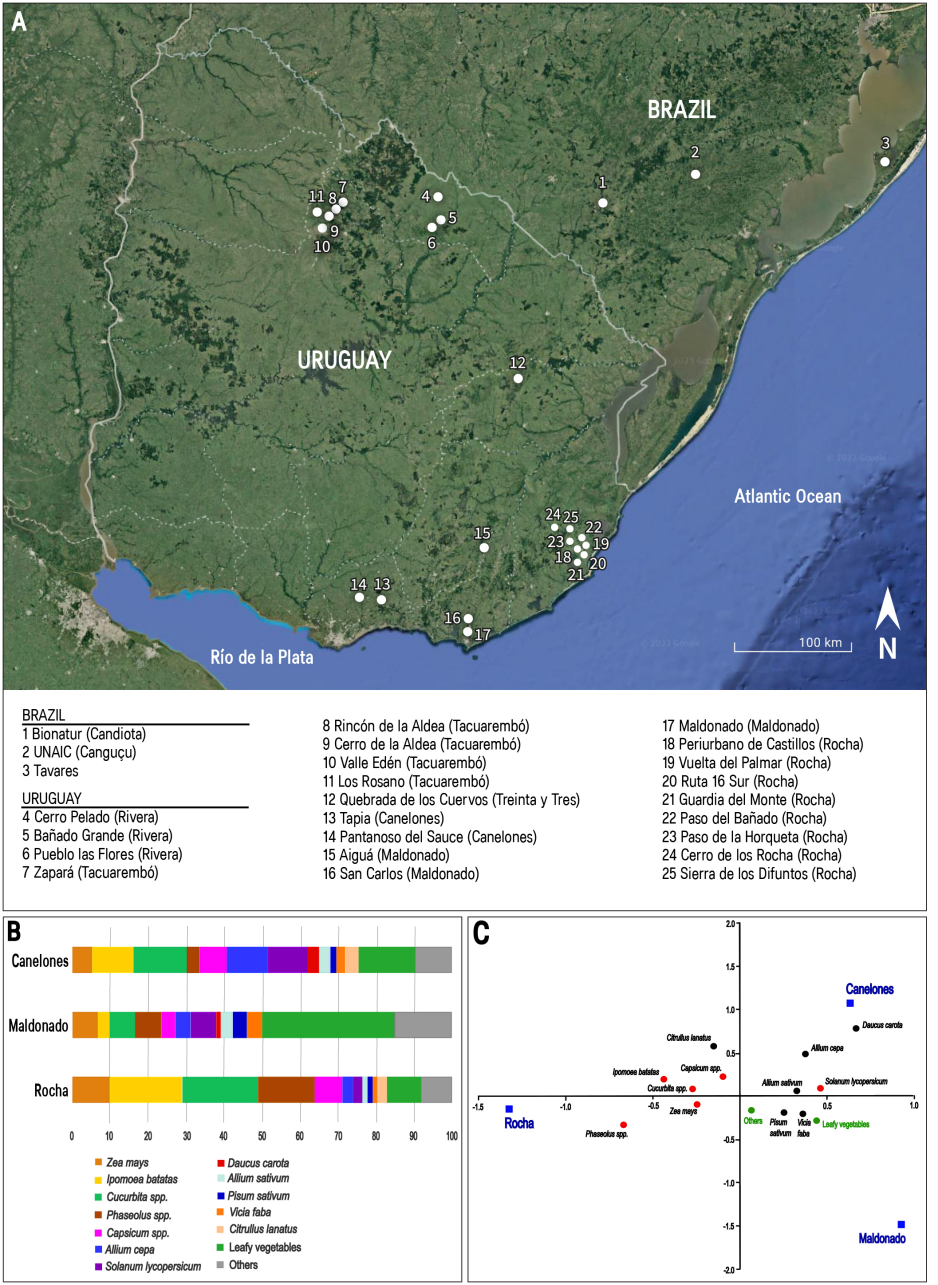
**(A)** Location of the territories with surveys of landraces conserved *in situ* (Design: Laura Moraiti); **(B)** Frequency of landraces by crop surveyed in the territories of Rocha, Maldonado and Canelones (Uruguay), based on Favaro and Piazza (2019); Pereira (2017) and Cuadro (2022); **(C)** Correspondence analysis for the distribution of landraces species in the territories of Rocha, Maldonado and Canelones.

**Figure 2A** presents the distribution of territories in which landraces and the agricultural systems were surveyed and farmers interviewed across Uruguay and southern Brazil. These surveys and interviews offer valuable insights into the productive systems and profiles of farmers who maintain this germplasm (Costa et al., 2010; Burgueño et al., 2015; Mello et al., 2017; Pereira, 2017; Favaro and Piazza, 2019; Vidal et al., 2021; Cuadro, 2022; Pádua et al., 2022). They shed light on the predominant vegetable crops, the associated traditional knowledge and practices, the various uses and purposes of production, and the farmers’ perceptions regarding the factors contributing to genetic erosion, as well as their motivations for landrace conservation.

#### 3.1.1 Landrace-conserving farmers and productive systems

The family farmers rooted in the territories for several generations act as the primary keepers of landraces. In more recent times, ‘neo-rural’ groups have also joined this endeavor like in ‘Maldonado’ territory, Uruguay (Cuadro et al., 2024). These are families which originate from urban cultures and have chosen to settle in rural areas (Calvário and Otero, 2015), and also have taken on the role of landrace keepers as a conscious commitment.

The main characteristics of family productive systems include residing on the farm, with the work primarily conducted by the family, and relatively small land areas (Piñeiro, 2004). The average sizes of each farm in the analyzed cases are approximately 20 hectares, and most of them are smaller than 50 hectares (Burgueño et al., 2015; Mello et al., 2017; Pereira, 2017; Favaro and Piazza, 2019).

These productive systems are agrobiodiverse, involving a combination of crops, livestock, dairy production, farm animals (pigs, poultry), fruit production, and non-productive areas rich in natural plant communities. In several cases, vegetable production is exclusively for self-consumption, with any surplus being sold (Burgueño et al., 2015; Pereira, 2017). However, many farmers have their main income from vegetables, as observed in ‘Tapia - Pantanoso del Sauce’ (Canelones, Uruguay) and in ‘Palmar de Castillos’ (Rocha, Uruguay), where horticulture constitutes the main activity (Pereira, 2017; Favaro and Piazza, 2019). Additionally, in many instances, the economy is supplemented by the production and sale of by-products such as sweets, jams, and preserves.

A notable aspect of family farming systems is the significant influence of generational succession on the stability of the production unit (Rodriguez-Lizano et al., 2020). The surveys revealed that traditional farmers are generally aged 60 years or older (Mello et al., 2017; Pereira, 2017; Favaro and Piazza, 2019). However, in certain regions like ‘Maldonado’ (Uruguay) ages are lower, with an average age of 48 for family farmers and 46 for neo-rural farmers (Cuadro, 2022). This difference may be attributed to the economic dynamics in ‘Maldonado’, providing better services and employment opportunities. When farmers were asked if they felt they had a generational replacement, 41% of them responded negatively (Favaro and Piazza, 2019). This issue is closely linked to the primary cause of genetic erosion in landraces.

From the surveys, as well, the fundamental role of women in the landrace conservation activities also arise, as well as the traditional knowledge on productive processes and uses that they hold. Although information on the agroecological management of landraces is usually lacking, some surveys indicated that approximately half of the farms practiced agroecology (Favaro and Piazza, 2019). In other cases, although not explicitly classified as such, the reliance on external inputs is relatively limited due to the traditional nature of these systems.

Farmers conserve landraces of various crops for several reasons, including adaptation or hardiness, organoleptic characteristics, culinary uses, post-harvest conservation, family tradition, heritage, food security, and economic importance (Vidal et al., 2021). Landraces enables them to maintain traditional or local knowledge, primarily passed down within families and shared locally, and which encompasses the characteristics, uses, and management practices associated with landraces.

#### 3.1.2 Crops and landraces

Landraces of 49 vegetable crops belonging to 47 species and 11 botanical families (excluding aromatic herbs) are currently cultivated in the Brazilian-Uruguayan Pampa biome (**Table 1**), as reported by Antunes et al. (2015); Barbieri et al. (2014); Cruz (2022); Fischer (2012); Fischer et al. (2016); Neitzke et al. (2015); Padilha (2017); Priori et al. (2022);. Priori et al. (2018); Priori et al. (2017); Burgueño et al. (2015); Pereira (2017); Mello et al. (2017); Favaro and Piazza (2019) and Cuadro (2022).

**Table 1:** List of landraces of vegetable species found in the Brazilian and Uruguayan Pampa, ordered by botanical family and edible organs.

Family	Common name	Scientific names	Edible organs
Alliaceae	Onion	*Allium cepa* L.	Bulbs
	Garlic	*Allium sativum* L.	Bulbs
	Leek	*Allium porrum* L.	False stem (leaf sheaths)
	Elephant garlic	*Allium ampeloprasum* L.	Bulbs
Amaranthaceae	Chard	*Beta vulgaris* var. *cicla* L.	Leaves
	Spinach	*Spinacia oleracea* L.	Leaves
Apiaceae	Celery	*Apium graveolens* L.	Leaves and petioles
	Carrot	*Daucus carota* L.	Roots
Asteraceae	Lettuce	*Lactuca sativa* L.	Leaves
	Chicory	*Cichorium intybus* L.	Leaves
Brassicaceae	Coles, Kale, Broccoli	*Brassica oleracea* L.	Leaves
	Turnip	*Brassica rapa* L.	Roots
	Mizuna	*Brassica rapa* L.	Leaves
	Mustard	*Sinapis alba* L.	Leaves
	Radish	*Raphanus sativus* L.	Roots
	Arugula	*Eruca sativa* Mill.	Leaves
	Squash and pumpkins	*Cucurbita argyrosperma* C.Huber *Cucurbita maxima* Duchesne *Cucurbita moschata* Duchesne *Cucurbita ficifolia* Bouché *Cucurbita pepo* L.	Fruits
Cucurbitaceae	Watermelon	*Citrullus lanatus* (Thunb.) Matsum. & Nakai	Fruits
	Bitter melon	*Momordica charantia* L.	Fruits
	Melon	*Cucumis melo* L.	Fruits
	Cucumber	*Cucumis sativus* L.	Fruits
	Chayote (vegetable pear)	*Sechium edule* (Jacq) Sw.	Fruits
	Gourds	*Lagenaria siceraria* (Molina) Standl	Fruits
	Sponge gourde	*Luffa cylindrica* (L.) M.Roem.	Fruits
Convolculaceae	Sweet potato	*Ipomoea batatas* (L.) Lam.	Tuberous roots
Euphorbiaceae	Cassava	*Manihot esculenta* Crantz	Tuberous roots
Fabaceae	Bean	*Phaseolus vulgaris* L. *Phaseolus lunatus* L. *Phaseolus coccineus* L.	Grains (seeds)
	Cowpea	*Vigna unguiculata* (L.) Walp	Grains (seeds)
	Grass pea	*Lathyrus sativus* L.	Grains (seeds)
	Faba bean	*Vicia faba* L.	Grains (seeds)
	Pea	*Pisum sativum* L.	Grains (seeds)
	Lablab	*Lablab purpureus* (L.) Sweet	Grains (seeds)
	Chickpea	*Cicer arietinum* L.	Grains (seeds)
	Peanut	*Arachis hypogaea* L.	Grains (seeds)
Poaceae	Maize	*Zea mays* L.	Grains (seeds)
Solanaceae	Peppers	*Capsicum baccatum* L. *Capsicum annuum* L. *Capsicum frutescens* L. *Capsicum chinense* Jacq.	Fruits
	Tomato	*Solanum lycopersicum* L.	Fruits
	Eggplant	*Solanum melongena* L.	Fruits
	Potato	*Solanum tuberosum* L.	Tubers (stems)

There are crop differences between Brazil and Uruguay, such as *Cucurbita argyrosperma*, *Capsicum frutescens*, *Manihot esculenta*, *Momordica charantia*, *Luffa aegyptiaca, Sechium edule*, and *Lagenaria siceraria*, which are only recorded in Rio Grande do Sul (Brazil). However, it is not ruled out that in future work they may also be recorded in Uruguay. On the other hand, in Uruguay, there are also records of landraces of crops such as *Lathyrus sativus*, *Lablab purpureus* and *Allium ampeloprasum*, which do not appear in the Brazilian records until now. This distinction could be influenced by cultural factors, and may also apply to regions within the countries, where differences in landraces adapted to different environments and management are expected.

For the more in-depth territorial surveys carried out by Pereira in ‘Rocha’ (2017), Favaro and Piazza in ‘Canelones’ (2019) and Cuadro in ‘Maldonado’ (2022) in Uruguay, 43 vegetable crops (40 species) belonging to 10 plant families and 426 landraces (cassava was notrecorded in these territories). Plant families more commonly found were Fabaceae, Cucurbitaceae and Brassicaceae, whereas the largest number of landraces corresponded to Cucurbitaceae (83), Fabaceae (71) and Solanaceae (60) (**Table 2**).

**Table 2:** Number of surveyed landraces in three territories in Uruguay by plant family, and diversity indices for the three territories.

Plant families	Number of surveyed landraces		
	Canelones	Maldonado	Rocha
Alliaceae	25	7	11
Amaranthaceae	6	7	3
Apiaceae	9	2	1
Asteraceae	4	6	2
Brassicaceae	18	23	6
Cucurbitaceae	35	10	38
Convolvulaceae	19	3	29
Fabaceae	17	22	32
Poaceae	9	7	15
Solanaceae	30	15	15
Diversity indices			
Species richness	32	28	29
Vegetable crops	32	31	29
Landraces richness	172	102	152
Shannon index	3.11	3.15	2.81
Equitability index	0.90	0.95	0.83
B index			
Canelones vs. Maldonado	<−−−− 0.83 −−−−>		
Maldonado vs. Rocha		<−−−− 0.70 −−−−>	
Canelones vs. Rocha	<−−−−−−−−−− 0.79 −−−−−−−−−−−−>		

From 426 maize and vegetable landraces in total, 172 were surveyed in ‘Canelones’ (32 species, 17 farms), 152 in ‘Rocha’ (29 species, 22 farms), and 102 in ‘Maldonado’ (28 species, 13 farms). The distribution among plant families, the Shannon -Wiener index, the evenness and Beta indices (**Table 2**) indicate high cultivated diversity, with differences in the number of landraces among the three territories. Landraces composition diversity for the most frequent crops and a correspondence analysis are shown in **Figures 2B, C**.

While landrace cultivation is confirmed, differences between territories due to commercial and cultural factors were observed. The main difference was observed between ‘Rocha’ and ‘Maldonado’. The region of ‘Rocha’ appears as productive systems based in traditional crops, mainly originated in America, related to self-consumption and to a lesser extent, to commercialization. A greater proportion of landraces of *Phaseolus* spp., *Cucurbita* spp., *Ipomoea batatas* and *Zea mays* than that in the other territories supports this characterization, also explained by a closer relation with farmers from South Brazil and the integration between vegetable cropping and animal production.

Surveyed territories in ‘Canelones’ and ‘Maldonado’ share features from either traditional household farming systems as well as a linkage with the markets. Leafy vegetables, tomato, carrot and onion landraces are important for the market supplying in Montevideo, capital of Uruguay, for ‘Canelones’, and the tourist area of Punta del Este in ‘Maldonado’. Especially in ‘Maldonado’, it is also observed the presence of non-traditional vegetables like mizuna, associated to neo-rural farmers. Nevertheless, both territories maintain landraces of traditional vegetables, particularly in ‘Canelones’.

Available information on *in situ* landraces conservation in the Brazilian-Uruguayan Pampa indicates, firstly, the relevant agrobiodiversity in the scant territories surveyed with comparable methodological data; and secondly, the need of generating an inventory allowing the identification and characterization of landraces with a high coverage.

#### 3.1.3 Farmer networks and associations linked to landraces conservation

Seed exchange among farmers is a common practice, often facilitated through landrace seed fairs and festivals organized by municipal and regional entities, civil society organizations, and local communities (Pádua et al., 2022). These events provide a platform for farmers to share seeds and knowledge about planting, management, harvesting, and storage of the landraces they cultivate. Additionally, seed exchange between neighboring farmers is prevalent and serves as a means of recovering lost seeds. In this way, conservation transcends individual properties, and collective conservation is a common practice (Louette et al., 1997; Hodgkin et al., 2007; Elteto, 2019; Fernandes, 2022).

In Uruguay, farmers engaged in landrace conservation often join networks such as the ‘Red Nacional de Semillas Nativas y Criollas’, the ‘Red de Agroecología’, rural development societies, cooperatives, local groups, NGOs, and land movements. The ‘Red Nacional de Semillas Nativas y Criollas’ is a civic national association with 35 local groups representing over 450 rural, suburban, and even urban farmers and social activists. They maintain approximately 300 landraces, including horticultural, agricultural, medicinal, aromatic, and native tree species. The network organizes the national native and landraces seed festival biannually, promoting the exchange of native seeds and landraces among farmers (Beltrán, 2020).

The project ‘National articulation for the governance and collective management of genetic diversity and its associated knowledge in family and peasant agriculture in Uruguay’ (Benefit-sharing Fund of the International Treaty on Plant Genetic Resources for Food and Agriculture - ITPGRFA) involves multiplication of landraces seeds of grass peas, squashes, peanuts, cowpea, and their participatory characterization. Some of these landraces will soon be made available through the ITPGRFA’s multilateral system.

In Brazil, a survey conducted by Embrapa in 2020 for the national report on plant genetic resources for food and agriculture highlighted the contributions in the Pampa biome from the NGO ‘Bionatur/Conaterra’ (Cooperativa Agroecológica Nacional Terra e Vida) and the ‘União das Associações Comunitárias do Interior de Canguçu’ (UNAIC) (Abreu et al., 2022). Bionatur is a seed producing organization of settled agrarian reform farmers, multiplying various species with a focus on vegetables. Bionatur maintains 80 vegetable landraces, and the seeds are preserved by a network of committing farmers, including 20 tomato landraces, 16 pepper, 4 carrot, 12 cucurbit, 4 lettuce, 2 chicory, 2 pea, and over 20 landraces of other species such as grains and flowers. UNAIC is a second-grade association that brings together various community associations and groups of family farmers. UNAIC advocates for the rights of family farmers and promotes sustainable rural development based on agroecological practices, supporting associationism and cooperativism. UNAIC maintains a collection of landraces, including 24 maize, 12 bean, 3 rice, 4 peanut, and 2 cowpea landraces.

### 3.2 *Ex situ* conservation

In a scenario of climate change, with the replacement of cultivated species, contamination by transgenes, and the reduction of human populations living in rural areas, the risk of landrace disappearance is increasing. Only an effective connection between *ex situ* conservation strategies and *in situ*/*on-farm* conservation can generate impactful results for the conservation and sustainable use of genetic resources. Both in Brazil and Uruguay, this integration between *ex situ* and *in situ*/*on-farm* conservation is still limited, but progress has been made.

Brazil has a germplasm collection and conservation system coordinated by Embrapa, in partnership with various public and private institutions. The most recent survey conducted (Abreu et al., 2022) recorded 370,066 samples of 2,330 species, belonging to 591 genera, stored in 177 genebanks. These genebanks are in 25 Embrapa units and 35 research institutions or universities throughout the Brazilian territory. Many landraces of vegetables have been collected and incorporated into the collections of these genebanks in the last two decades. Much of this material has already been characterized by using morphological descriptors and molecular markers, undergoing agronomic evaluation and identification of bioactive compounds. A backup copy of the samples from genebanks of species with orthodox seeds is kept in cold rooms at Embrapa’s Genebank in Brasilia. A second backup copy of some of these samples is deposited in the Svalbard Global Seed Vault, in the Arctic Circle. Among the 5,122 accessions deposited by Embrapa in Svalbard, there are several landraces of peppers, squashes, melons, watermelons, and onions collected over the past two decades in the Brazilian Pampa biome (Embrapa recursos Genéticos e Biotecnologia, 2023). The data from Brazil’s genebanks are stored in the ‘Alelo System’ (Souza et al., 2022), a set of software designed to document, computerize, manage, and handle data and information generated in genebank activities. The information from ‘Alelo’ is transmitted to ‘Genesys’, an international database on genetic resources related to food and agriculture, at least three times a year.

*Ex situ* conservation in Uruguay is carried out by the Instituto Nacional de Investigación Agropecuaria (INIA) and the Facultad de Agronomía, Universidad de la República (BG-Fagro). In the last decade, the number of systematic collections associated with different projects has expanded, and the new accessions have been characterized, including ethnobotanical information to enhance passport data (Comité Nacional de Recursos Fitogenéticos, 2021). Together, both institutions conserve over 26,000 accessions (Comité Nacional de Recursos Fitogenéticos, 2018), with INIA having the largest collection (Condón and Rossi, 2018). The main collections of landraces consist of maize with 950 accessions, while the collections of beans, peanuts, onions, carrots, peppers, tomatoes, and squashes have less than 100 accessions each, collectively accounting for less than 10% of the accessions conserved *ex situ*.

Through the project ‘National Articulation for the Governance and Collective Management of Genetic Diversity and Associated Knowledge in Family Farming and Peasant Agriculture in Uruguay’ funded by the fourth call of the Benefit-Sharing Fund of the International Treaty on Plant Genetic Resources for Food and Agriculture (ITPGRFA), four community banks have been established. These banks, called Regional Community Centers for Agrobiodiversity Support (CCRA), are managed by farmers to facilitate access, storage, distribution, multiplication, and participatory improvement of landraces of 15 species (FAO BSF Project, 2021).

Community Seed Banks (or Seed Houses) in southern Brazil also deserve recognition as popular initiatives managed by groups of farmers with the objective of conserving landraces and ensuring access to seeds (Abreu et al., 2022). These activities constitute community biodiversity management practices, promoting resilience (Boef et al., 2013).

In Brazil, the project ‘*In Situ/On-Farm* Conservation of Plant Genetic Resources and its interaction with *Ex Situ* Conservation/ConservaIn’ is led by Embrapa and involves several universities, NGOs, farmers’ associations, and seed guardians. The project’s activities are carried out in all Brazilian biomes and aim to systematize and make available information on *in situ* – *on farm* conservation actions of plant genetic resources. An interactive database is ongoing, where all the participants can enter data on the maintained landraces, indicating threats and the context of conservation and use of this germplasm. This database will allow the identification and mapping of landraces, enabling a diagnosis of on-farm conservation and facilitating the development of maintenance strategies while minimizing genetic erosion. Surveying data on traditional people and communities whose agricultural systems contribute to the existence of microcenters of local diversity of cultivated plants contribute to valorize and recognize territories and local culture that preserve this diversity (Pádua et al., 2022).

In both countries, *ex situ* collections are not sufficient, and there is a need for systematic collections, monitoring of conservation status, and increased characterization activities. Germplasm collection actions in partnership with seed guardians and community seed banks can bring visibility to the role of guardians, contributing to their empowerment and promoting the integration between *ex situ* conservation strategies and *in situ*/*on farm* conservation.

### 3.3 Case studies

#### 3.3.1 Onion landraces in Uruguay

**Figure 3:**
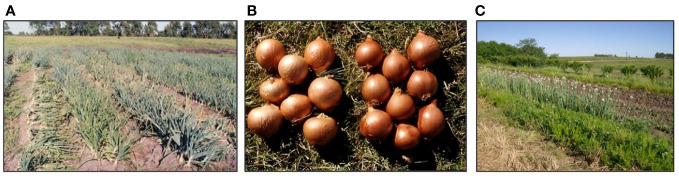
Onion landraces. **(A)** Comparative agronomic field trial of onion landraces showcasing differences in harvesting time (top-down), leaf color, and foliage habits. **(B)** Example of a typical intermediate-day landrace population with a light brown colour (left) and a long-day (late) landrace population with a dark brown skin color (right). **(C)** Illustration of a typical seed-producing row in small-scale household family farming systems.

Onions (*Allium cepa* L.) are the third vegetable crop after potatoes and tomatoes in Uruguay in terms of Gross Production Value, and the most relevant in the number of vegetable farmers involved (MGAP-DIEA (Ministerio de Ganadería, Agricultura y Pesca - Dirección de Investigaciones Económicas Agropecuarias), 2020). The maintenance of onion landraces in small household farms is a traditional practice, leading to a reservoir of an important genetic diversity (e.g., harvesting time, bulb type, color and shape, post-harvest conservation and consumer uses (Galván et al., 2005) (**Figure 3**). Onions remain as one of the main vegetable crops in Uruguay with landraces having a great economic relevance and share in the main vegetable markets. However, *in situ* conservation of onion genetic diversity has decreased and is under risk. Genetic erosion is mainly due to the decreasing number of farmers and a general rural-to-urban migration, as well as the replacement of landraces by improved national cultivars grown by the new generations of farmers.

Onion landraces prospection and collections were performed in the first period between 1987 and 1991 (Galván, 1993; Galván et al., 1997) in ‘Canelones’, the main vegetable production area. A subsequent collection effort in 1997 and 1998 covered the whole country (González and Vilaró, 1999). In total, about 60 onion accessions were collected and conserved as a working collection at the BG-Fagro. Through productive evaluations, it was discovered that onions exhibit a wide range of genetic diversity in terms of harvesting time, with diverse photoperiod requirements for bulbing onset, which can be seen as an important adaptive trait (Galván et al., 1997). Initial agronomic comparisons strongly suggest that intermediate-day (ID) onion landraces, harvested in December, exhibit the highest yield potential for southern Uruguay (Galván, 1993). Notably, there were no introduced cultivars with a similar growing cycle available at that time, emphasizing the significance of this discovery. Additionally, onion landraces differed in postharvest conservation, a crucial economic trait that enables market supply for six to eight months after harvest (Galván et al., 1997; González and Vilaró, 1999).

**Figure 4:**
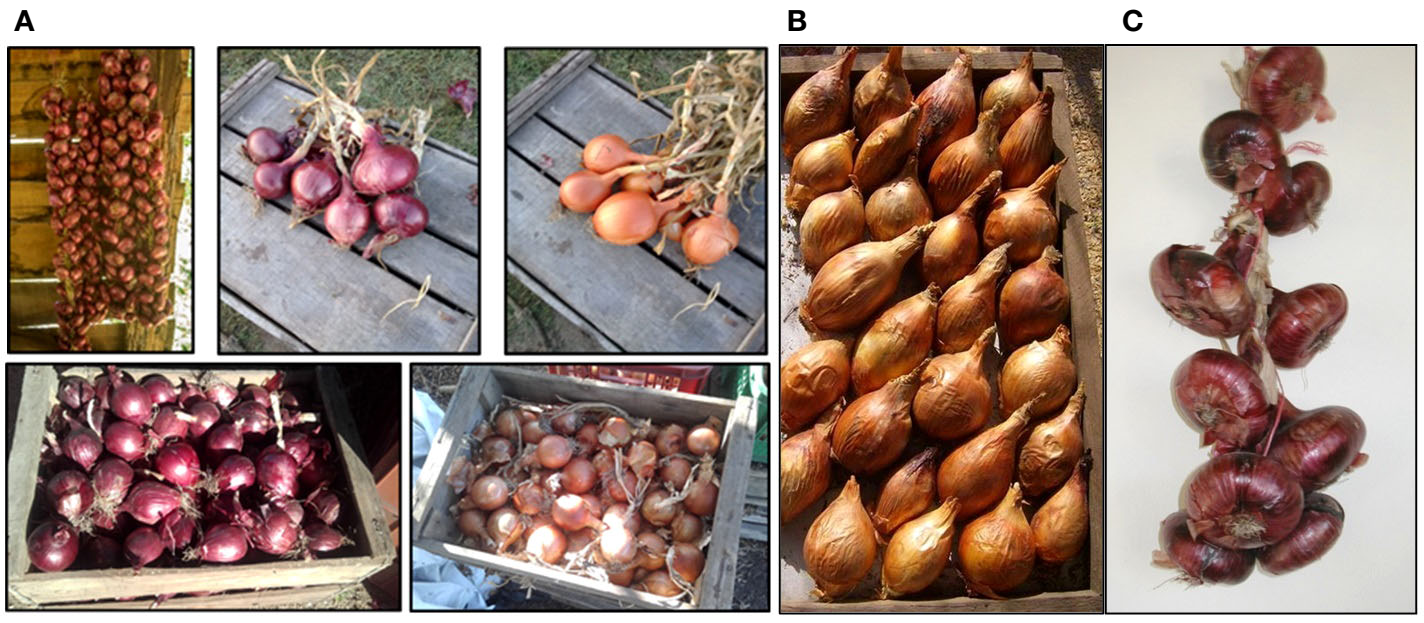
Onion diversity for bulb characteristics. **(A)** onion landraces and postharvest conservation (Source: Favaro and Piazza, 2019); **(B)** onion landrace with elongated bulb shape or bottle shape achieved through intentional selection; **(C)** red flat onion landrace, marketed as bunches of entire bulbing plants or as dry bulbs.

Onion landraces in Uruguay exhibited variations in traits such as leaf color and foliage habit (ranging from erect to prostrated), as well as in their response to leaf diseases such as white spots caused by *Botrytis squamosa* (Galván et al., 2004) and mildew caused by *Peronospora destructor* (Colnago et al., 2012). The landraces also displayed diversity in bulb quality traits, both within and between landraces, including variations in skin color, number of skin layers, bulb shape, and neck thickness (Galván et al., 1997; González and Vilaró, 1999) (**Figure 4**). A phenotypic evaluation conducted by Porta et al. (2014) on lines derived from an onion landrace highlighted significant genetic diversity in leaf length, bulb and neck diameters at harvest, bulb weight, soluble solids, and dry matter content within the original population and derived lines.

Monteverde et al. (2014) conducted a study on genetic diversity based on ISSR molecular markers, comparing 27 onion landraces and two national improved cultivars derived from former landraces. The study revealed that most genetic diversity exists within populations (66%), whilst the variation among populations represents a smaller share of the total genetic diversity. Distinct molecular markers were observed for early landraces (short day onions), whereas intermediate and late landraces (long day onions) formed a unified group without clear differentiation. Both intermediate and late landraces are cultivated in Canelones. Interestingly, even red skin onion landraces, which is a trait controlled by a few genes, were grouped together with brown intermediate- and long-day landraces. Harvesting time and phenotypic traits, such as a lighter brown color for the intermediate onions and brown to dark brown for late onion landraces, allowed easy phenotypical distinction between intermediate and late landraces. However, these differences were not reflected by the neutral ISSR markers. These findings are likely attributed to inter-pollination between nearby seed-producing plants, as well as intentional mixtures between landraces aimed at improving quality traits (Monteverde et al., 2014). Typically, family farmers have traditionally retained and continued to use their own seeds, often passed down through generations from their parents or grandparents. However, they may choose to blend their landrace populations with other landraces and cultivars if they believe the mixture will enhance agronomic and quality traits (Galván et al., 2005).

Onion breeding was initiated in the 1980s by governmental institutions in Uruguay, leveraging the genetic diversity present in landraces. These efforts resulted in the development and release of a series of locally improved cultivars (Vicente et al., 2010). Notably, the cultivars ‘Pantanoso del Sauce CRS’ and ‘INIA Casera’ gained significant popularity among growers, with their adoption surpassing 50% of the cultivated area in the south and north vegetable regions, respectively.

The rapid adoption of locally developed cultivars was closely tied to the establishment of a certification seed production program, a novel venture within the Uruguayan vegetable sector. Some family farmers formed a cooperative seed production enterprise, creating a new business opportunity through a collaborative effort between government and private entities (Peluffo et al., 2016a).

The seed certification program created opportunities for research in seed production technology, encompassing various aspects of crop protection and management (González et al., 2011; Peluffo et al., 2016b). Thanks to the genetic richness of onion landraces, improved cultivars enabled their competitiveness in major vegetable markets. The current coexistence of diverse seed systems is acknowledged in the literature (Lammerts van Bueren et al., 2018). Considering the interactions between formal breeding and traditional seed systems, a revised and broader concept of landraces was proposed by Casañas et al. (2017), under which landraces may be improved, but they may still be considered evolved landraces because of their main genetic background.

#### 3.3.2 Forty centuries of maize in Uruguay

**Figure 5:**
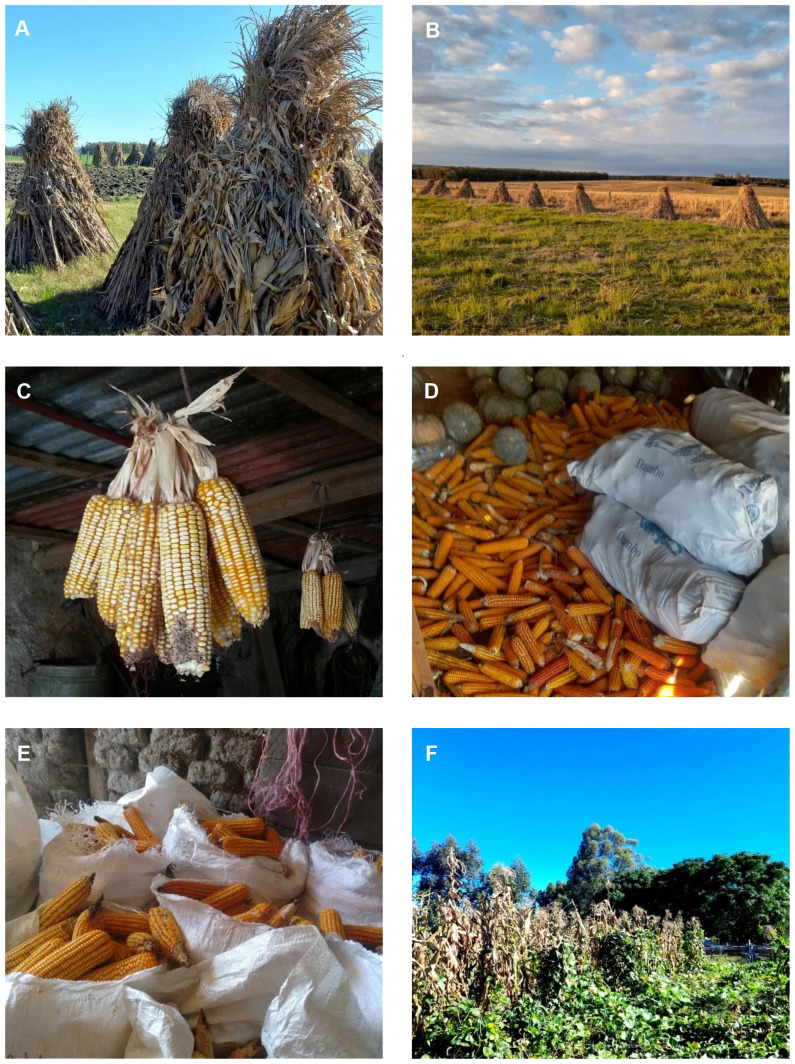
Local family systems of maize production and conservation in Uruguay. **(A)** Maize conservation system in parvas or pirvas, Canelones department, **(B)** Maize conservation system in parvas or pirvas, Tacuarembó department, **(C)** Maize conservation system by hanging in Rocha department, **(D)** Maize conservation system in ears in Rivera department, **(E)** Saize conservation system in ears in Canelones department, **(F)** Crop consortium of corn, beans and cassava, in Rivera department (Source: InterABio 2018, 2021, 2022).

Maize holds the position of the third most important rainfed cereal in Uruguay, following wheat and barley (MGAP-DIEA (MGAP-DIEA (Ministerio de Ganadería, Agricultura y Pesca - Dirección de Investigaciones Económicas Agropecuarias), 2020). It is considered a traditional crop with three main purposes, to produce grain, to create fodder reserves for animal productive systems, and albeit on a smaller scale, it is cultivated for horticulture and self-consumption in small areas of family production, relying on landraces and minimal input usage (**Figure 5**). Additionally, a portion of traditional maize production in small areas is processed in artisanal industries and sale in local markets that place value on the origin of these products.

Maize cultivation in the Pampa biome has a long-standing agricultural and cultural tradition. One of the earliest documented references in the early 19th century are the writings of Pérez Castellanos (1968), identifying four types of maize: ‘Blancos’, ‘Minas’, ‘Morocho Canario’, and ‘Morocho Rojo’. These types are distinguished by their grain type and purpose of use. The ‘Blanco’ possessed floury grains used for baked goods, while the ‘Minas’ had a popcorn pointed grain type, usually popped in hot cow fat. The hard landraces ‘Morocho Canario’ and ‘Morocho Rojo’ were used to make ‘mazamorra’ and for medicinal use. Paterniani and Goodman (1977) classified maize of South American lowlands into four groups based on age, origin, and endosperm type. The indigenous group consisted of maize with floury and *pisingallos* endosperm types (popcorn), whilst the ancient commercial maize group possessed hard endosperm. The modern landraces had dentate and semi-dentate endosperms. Maize dispersion in South American lowlands involved migratory flows from the Amazon region (Kistler et al., 2018), particularly from ethnic groups of Macro-Jé, Arawak, and Tupi-guaraní linguistic roots. Two possible routes that include the Pampa biome as part of the dispersion process were indicated by Costa et al. (2022). These routes explain the main contributions to the current diversity, the first would contribute floury maize with a possible origin in the southwest Amazonia, and a second route occurred along the Atlantic Coast, bringing hard, floury, and *pisingallo* maize to the South. The different migrations that have occurred in recent centuries have brought other types like dent and semi-dent corn that have been included in the crops and cultures of the Pampa biome.

In the 1970s, the Uruguayan maize collection was established to conserve landraces *ex situ*, comprising 859 landraces with a predominance of orange kernels and flint type (Fernández et al., 1983). This collection was characterized (De León et al., 1978), and later reclassified by De María et al. (1979).This new classification identified ten races, including a floury grain race ‘Moroti’ and a popcorn race ‘Pisingallo’ considered indigenous. The other races were considered as introduced: flint grain, dent or semi-dent grains.

The comparative distribution of the genetic diversity of maize collections in the Southern Cone allowed identifying Uruguay as a region with the greatest diversity of grain types (Vilaró et al., 2020). Proposals for core collections based on phenotypic diversity highlighted the diversity between and within races (Malosetti and Abadie, 2001; Ozer-Ami et al., 2004), whereas SST markers identified four genetic groups within the ‘Blanco Dentado’ race (Porta et al., 2018). These data consistently point to diversity within races, even for modern introduced landraces.

**Figure 6:**
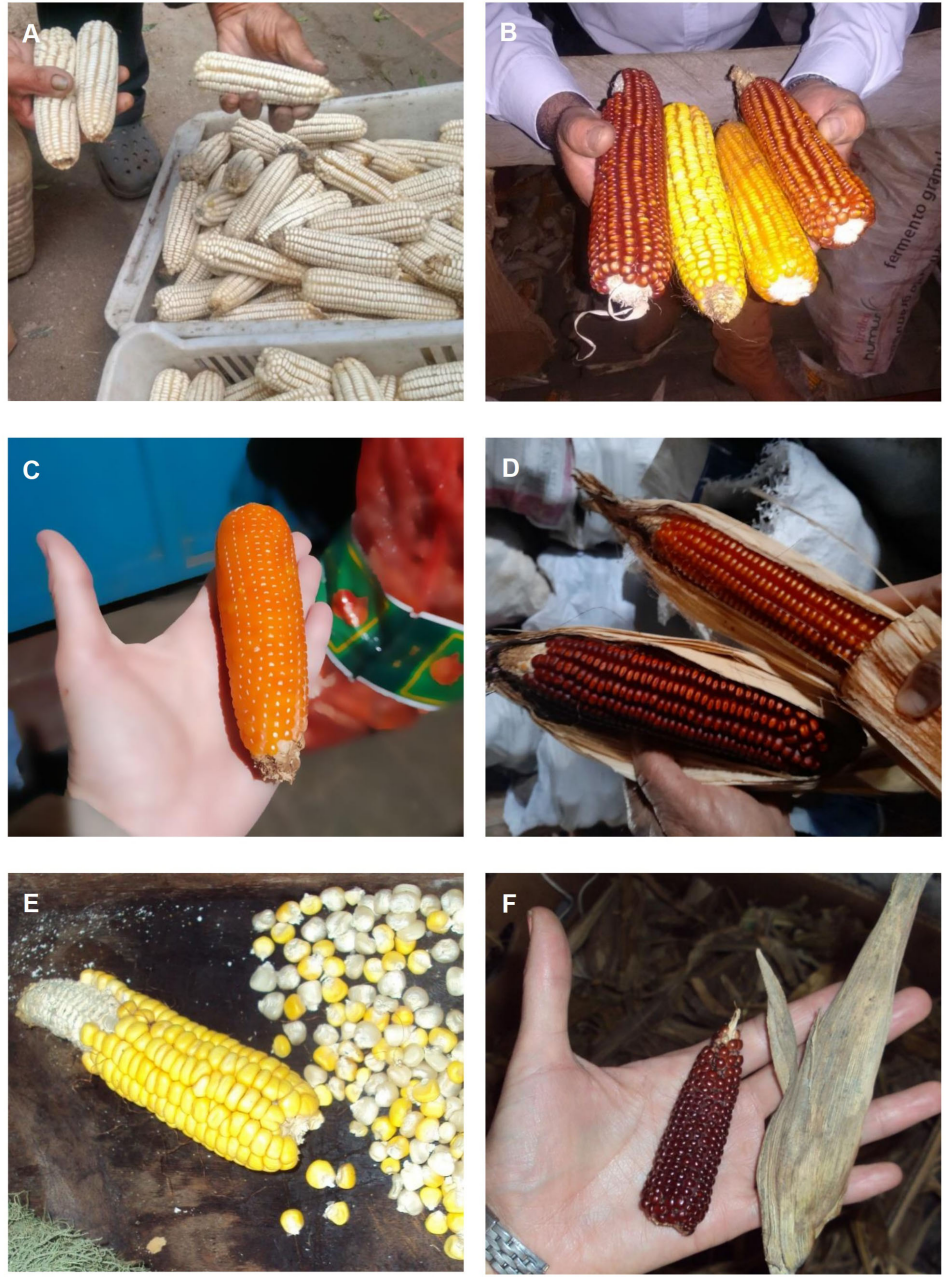
Maize diversity. **(A, B)** Dent, **(C, D)** Flint, **(E)** Floury, **(F)** Popcorn (Source: InterABio, 2018, 2021 y 2022).

Since the 1970s, a decrease in the number of families preserving maize in Uruguay has been observed, due to generational turnover and rural production abandonment (Achkar et al., 2017). While in the late 1970s one every two farmers saved their own seeds (Cibils et al., 1995), thirty years later it is estimated that only one out of 77 landraces remain in cultivation (Vidal et al., 2011). In order to update the knowledge of *in situ on-farm* conserved maize landraces in Brazil and Uruguay, 138 families from both countries were interviewed (Silva et al., 2020b). In Uruguay, 38 families were visited, and samples of 79 landraces were collected in the north, east, south, and northwest regions (**Figure 6**). The richness of local names varied by region, ranging from 2 in the northwest to 8 in the south, 15 in the north, and 22 in the east. Landrace names serve as indicators of landrace knowledge and reflect the diversity of types and uses. Samples of the landraces were used to create a collection preserved at the BG-Fagro for morphological and molecular marker characterization. Across the four regions, most of the identified landraces had dentate and semi-dentate kernels (40%), followed by hard and semi-hard kernels (39%), while floury kernels (15%) predominated in the north and south. The study of 69 of these landraces using Single Nucleotide Polymorphisms (SNPs) molecular markers indicated that the diversity between the north, east, and south regions is currently higher than within them (Vidal et al., 2019). Based on this work, an update of the Brazilian and Uruguayan races was proposed, considering their origin, endosperm type, grain, and ear characteristics (Silva et al., 2020a). The classification identified 10 races or race complexes for the Pampa biome, including Uruguay: ‘Avati Morotí’, ‘Caingang’, ‘Morotí Caingang’ Complex, ‘Cateto Sulino’, ‘Cateto Sulino Groso’, ‘Cateto Sulino Groso’ Complex, ‘Cristal’, ‘Amarillo Dentado’, ‘Blanco Dentado’, and ‘Pisingallo Redondo’ (Silva et al., 2021). The ‘Caingang’ and ‘Pisingallo’ Redondo races were not included in De María et al. (1979) classification, and no pointed popcorn was found, indicating the dynamic nature of landraces, with some disappearing and new ones emerging (Perales and Golicher, 2014).

As a tool to promote the conservation of maize landraces, Costa (2020) developed a methodology to identify microcenters of diversity for Brazil and Uruguay using ethnobotanical, morphological, and molecular data from the collection preserved at the BG-Fagro. The authors considered three criteria: richness of landraces exclusive to each region; socio-cultural aspects (such as uses, management, seed origin); and ethnobotanical, phenotypic, and molecular indices. Based on these criteria, microcenters of diversity were proposed for the eastern, northern, and southern regions of Uruguay. Microcenters of diversity for cultivated species are associated with geographically restricted areas where significant diversity accumulates (Harlan, 1971; Harlan, 1992).

Recent studies have reported advantages of landraces over commercial hybrids in terms of susceptibility to diseases, presence of endophytes, and rhizosphere bacteria. Álvarez (2022) identified differences of 3% to 43% in the levels of ear infection by *Fusarium verticillioides* and *F. proliferatum*. Trasante et al. (2022) found significant differences in the endophytic microbiome of seeds from 12 landraces, and Azziz et al. (2021) isolated 29 strains of bacteria from the rhizosphere and soils associated with maize landraces. These isolated endophytes and bacteria have potential as fungal inhibitors, plant growth promoters, nitrogen fixers, and phosphorus solubilizers.

Two examples of the potential of landraces for genetic improvement are the races ‘Blanco Dentado’ and ‘Pisingallos’. The breeding program of the ‘Facultad de Agronomía’ (Universidad de la República, Uruguay) started with the collection made by De León et al. (1978), which, after a selection process involving the participation of farmers (Alesandri, 2012), resulted in the formation of a ‘Blanco Dentado’ racial composite in 1981 (Abadie et al., 1997). From this composite, the ‘Blanco Cangüé’ cultivar was selected, which exhibited high productivity (Padrón and Lust, 1986), fodder quality (Larghero, 2011; Alesandri, 2012), and adaptation (Medina et al., 2001; Vidal et al., 2009).

In the case of ‘Pisingallos’ the objective is to reintroduce white-pointed maize, which was common in the last century (Fernández et al., 1983) and lost in the present. As a way to value this lost race, a project was developed in collaboration with the school gardens program (Silveira et al., 2019). The multiplication and evaluation of five accessions from the *ex situ* collection conserved by INIA was established in two schools. The students actively participated in these areas of multiplication, after they conserve seeds and the popped grains were consumed in schools (Silveira et al., 2018; Cabrera et al., 2020).

Research on lowlands maize highlights the importance of landraces in the Pampa biome and the efforts of family farmers in their conservation. The historical significance of cultivation, the presence of microcenters of diversity, and the abundance of landraces with direct and potential use, call for joint actions to safeguard this genetic and cultural heritage.

#### 3.3.3 Landraces of Cucurbitaceae in the Brazilian and Uruguayan Pampa biome

In the Brazilian and Uruguayan Pampa biom*e*, farmers cultivate a large number of Cucurbitaceae landraces, with traditional knowledge associated on crop management practices, selection methods, seed storage, and related local recipes and histories (Barbieri et al., 2014; Rivas et al., 2018). Farmers select their own seeds based on different criteria such as fruit skin color, fruit shape and size, pulp texture and flavour (Priori, 2015). Usually, seeds are preserved in a range of containers, such as plastic bottles (PET) and glass jars.

In a survey conducted in the territory of ‘Palmar de Castillos’ (Department of Rocha) in Uruguay, it was observed that more than a third of the landraces has been managed by families for more than 30 years: 48% of the surveyed rural families conserved landraces of squash, watermelon, melon and cucumber. Thirty-eight landraces of six different species were identified (14 *Cucurbita moschata*, 18 *Cucurbita pepo* and *Cucurbita maxima*; 4 *Citrullus lanatus* and 2 *Cucumis melo*). These landraces have been traded between relatives or neighbors or constitute a family heirloom, where seeds pass from generation to generation (Rivas et al., 2018).

In Brazil, according to the data available in the Sistema ‘Alelo Vegetal’ – the database of genetic resources of Embrapa (Embrapa recursos Genéticos e Biotecnologia, 2023) –from 2002 to 2019, 285 landraces were collected in the Pampa biome for conservation in the Cucurbitaceae Genebank of Embrapa Clima Temperado (Pelotas, Rio Grande do Sul). These landraces include watermelon (*Citrullus lanatus*), melon (*Cucumis melo*), cucumber (*Cucumis sativus*), bottle gourd (*Lagenaria siceraria*), sponge gourd (*Luffa aegyptiaca*) and the five domesticated species of squash and pumpkins (*Cucurbita argyrosperma, Cucurbita ficifolia, Cucurbita maxima, Cucurbita moschata* and *Cucurbita pepo*).

*Cucurbita* landraces are widely used for food, depending on the species and the ethnic origin of the local communities. The largest number of landraces in cultivation in the Brazilian Pampa are the species *Cucurbita maxima* and *Cucurbita moschata*. The former presents great genetic variability for external morphological fruit traits, such as size, shape, color and shell texture, which results in a wide range of local names given to each type. The fruits of *Cucurbita moschata* represent an important food reserve for domestic animals (mainly swines and bovines), as well as being widely used in sweet recipes and also in savory dishes (purées, soups and stews). Farmers descending from Portuguese immigrants who arrived in the Brazilian Pampa in the 19th century still grow *Cucurbita pepo* whose fruits, known as *mogangos*, have a fairly hard shell and fibrous pulp, and are appreciated in the preparation of ‘caramelized mogango’ and also in savoury dishes. In much smaller numbers, some farmers also keep landraces of ‘cidra’ or ‘gila’ (*Cucurbita ficifolia*), with extremely hard hulled fruits and white and very fibrous pulp, used in the preparation of a typical Portuguese sweet dish called ‘doce de gila’ (Barbieri, 2012; Barbieri et al., 2014; Priori, 2015; Priori et al., 2017; Priori et al., 2018). Landraces of Cucurbita argyrosperma are quite rare. The few farmers who maintain them reported that their cultivation was much more common more than 40 years ago.

Afro-descendants have their own landraces, particularly those living in *quilombola* communities, mainly ‘mogangos’ (*Cucurbita pepo*), as well as landraces of *Cucurbita maxima* and *Cucurbita moschata*. The descendants of German and Pomeranian immigrants, in addition to *Cucurbita maxima* and *Cucurbita moschata*, keep ornamental landraces of *Cucurbita pepo*, some with edible fruits and others that are not suitable for consumption due to the bitterness of the pulp. With intense and quite varied colours, as well as quite diverse formats (periform, oval, discoid, round, star-shaped), the fruits are used in home decoration. The so-called ornamental *poronguinhos* have piriform format, are quite varied and can be used in decoration, with great post-harvest conservation. When they are star-shaped, they are called *star squash* or *abóbora-dez-mandamentos*, and are used in sweet preparations, with or without the addition of shredded coconut (Barbieri, 2012). Considering that the content of bioactive compounds, antioxidant activity and concentration of minerals (iron, potassium, calcium, phosphorus and copper) varies widely in the pulps of different squash landraces (*Cucurbita maxima* and *Cucurbita moschata*), some landraces can be used to develop biofortified cultivars aiming to promote consumer health (Priori et al., 2017; Priori et al., 2022).

**Figure 7:**
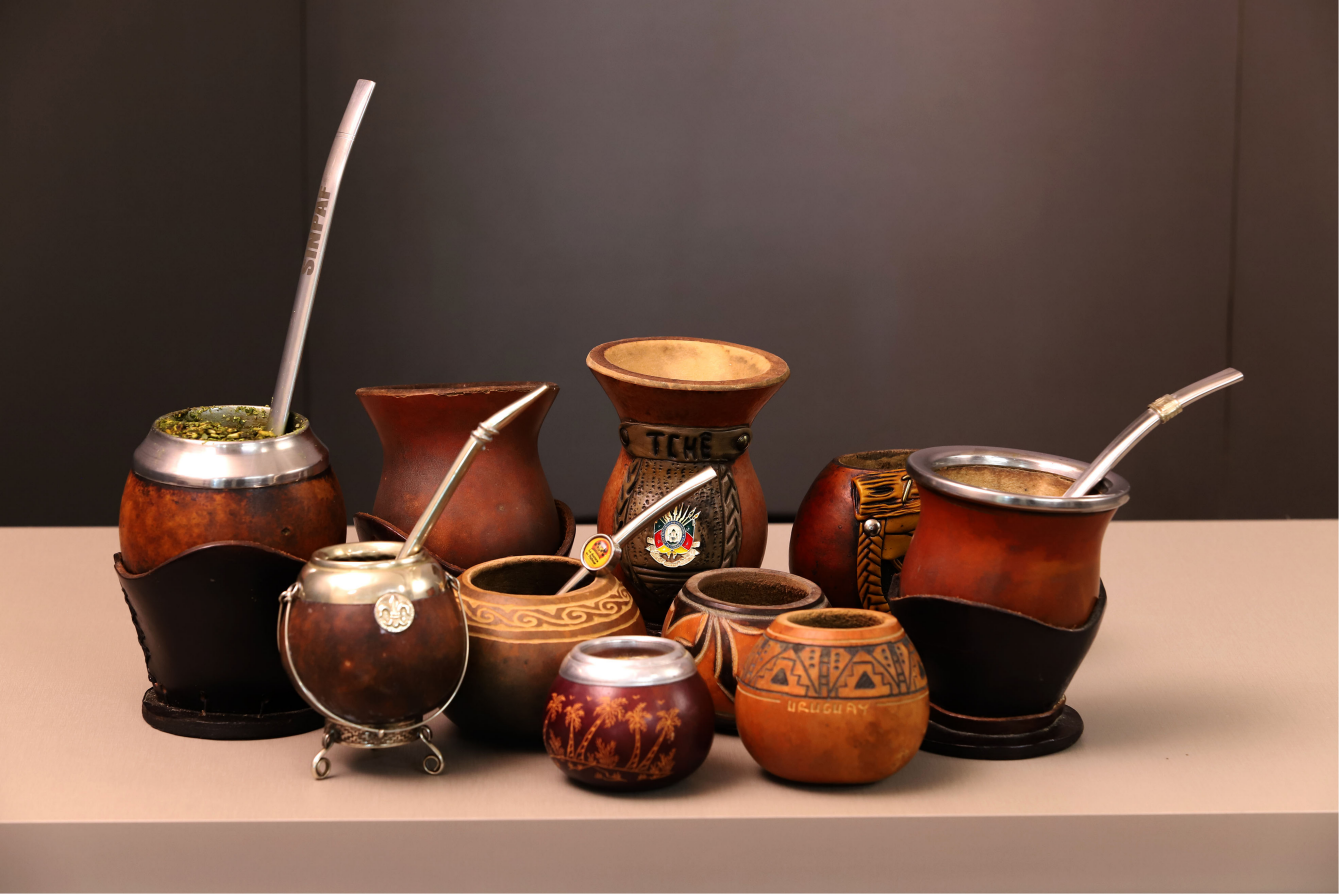
Mate gourds made with landraces of *Lagenaria siceraria* from Uruguay and Brazil. (Picture: Paulo Lanzetta).

The fruits of *Lagenaria siceraria* landraces are used to make the *cuias de chimarrão* or *mates* (**Figure 7**), which are drinking vessels (gourds) used for drinking *mate* or *chimarrão*, a traditional beverage of the Pampas made with leaves of *Ilex paraguariensis* (Aquifoliaceae). In turn, the fruits of the sponge gourd (*Luffa aegyptiaca*) are used in the Brazilian Pampa as a sponge for washing dishes or for bathing.

**Figure 8:**
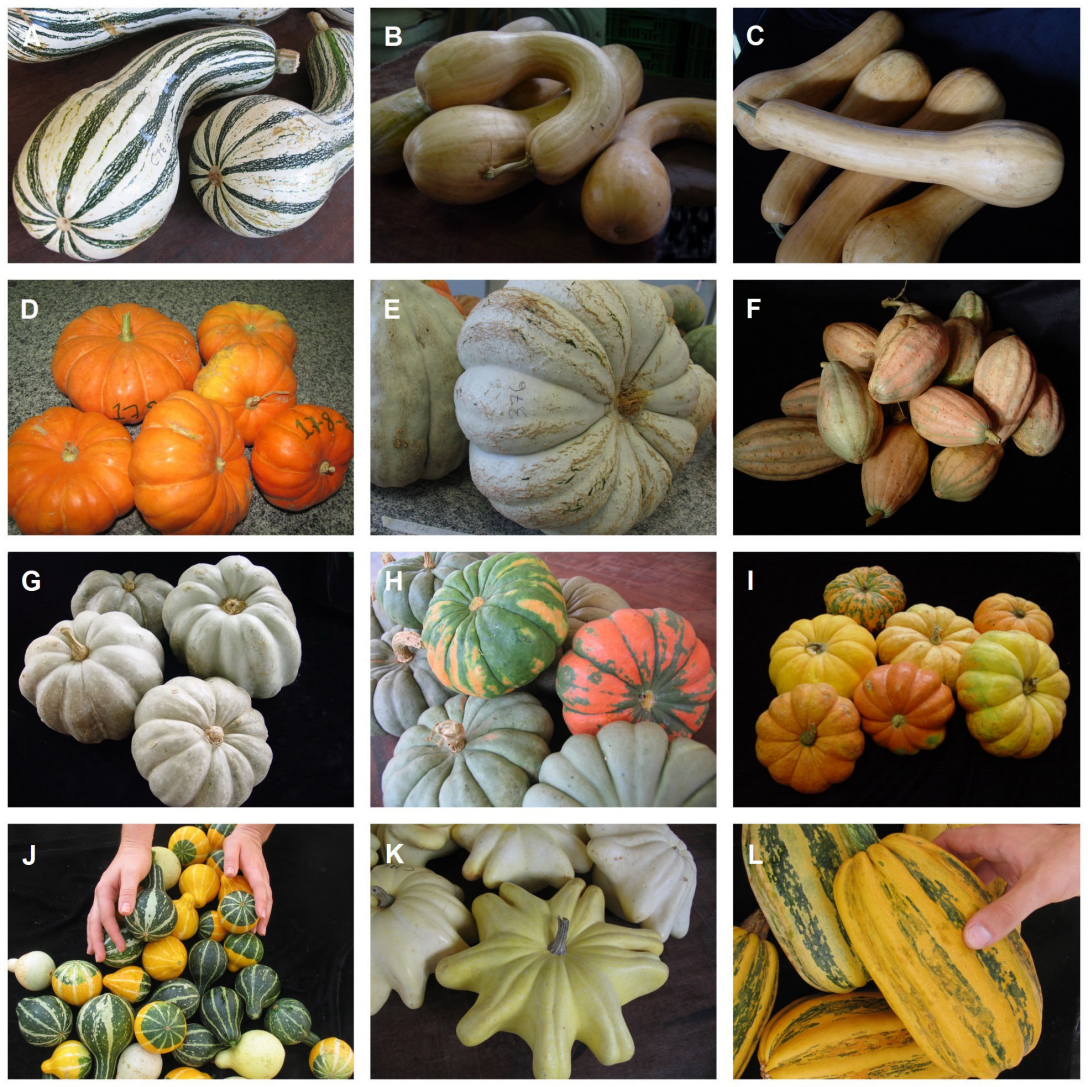
Landraces of Cucurbita cultivated by farmers in the Brazilian Pampa biome: **(A)** *Cucurbita argyrosperma*, **(B, C)** *Cucurbita moschata*, **(D–I)** *Cucurbita maxima*, **(J–L)** *Cucurbita pepo*. (Pictures: Rosa Lía Barbieri).

Based on the characterization and evaluation of the *Cucurbita* accessions of the Genebank of Embrapa Clima Temperado, we conclude that Brazil is a secondary center of *Cucurbita* diversity. The greatest genetic variability is found in the south of the country, especially in the Pampa biome (**Figure 8**). This has been corroborated by the results of morphological and molecular characterization with microsatellite markers, and characterization of bioactive compounds, antioxidant activity and mineral contents (Fischer, 2012; Priori et al., 2012; Priori et al., 2013; Fischer et al., 2015; Fischer et al., 2016; Priori et al., 2017; Valduga, 2017; Priori et al., 2018; Priori et al., 2022).

#### 3.3.4 *Capsicum* peppers

*Capsicum* peppers (Solanaceae) have the presence of capsaicin in their fruits as a distinctive feature. Originating in the Americas, these peppers became known worldwide after the voyages of Christopher Columbus in the 15th century (Barbieri and Neitzke, 2008). *Capsicum* peppers are part of the cultural wealth and valuable heritage of South American agrobiodiversity. They are cultivated in an immense variety of types, names, sizes, colors, flavors and pungencies (Henz and Costa, 2005). Brazil is an important secondary center of diversity for domesticated species of the genus *Capsicum*, with considerable genetic variability in *C. annuum, C. baccatum, C. frutescens* and *C. chinense* (Reifschneider, 2000). The uses of *Capsicum* peppers are diverse and related to local traditions and the availability of landraces in cultivation regions (Cruz, 2022). They are used in the preparation of various dishes, consumed *in natura* or processed into sauces, jellies, preserves, dehydrated, and are used as ingredients in pharmaceutical products and personal defense weapons (pepper spray). Due to their aesthetic characteristics, they are also cultivated as ornamental plants.

In the Pampa biome, many families maintain landraces of *Capsicum* peppers. Some people grow a few plants, produced in pots or planted in their domestic or vegetable gardens for various uses, such as in cooking and as ornamental plants (Neitzke et al., 2016), but there are also farmers who grow them in commercial plantations (Neitzke, 2012; Favaro and Piazza, 2019; Cruz, 2022). As in various regions of the world, in the Pampa biome the *Capsicum* genus peppers are used as ingredients in typical dishes, such as *entrevero* (consumed in southern Brazil and Uruguay), *feijoada* (consumed mainly in Brazil) and *chimichurri* (a sauce consumed in Argentina, Uruguay and southern Brazil).

In the Brazilian Pampa, the municipality of ‘Turuçu’ is outstanding in the cultivation of peppers. It is recognized as the Brazilian capital of red peppers, also known as red finger peppers (*Capsicum baccatum*). Commercial crops have been produced for about a century, on small rural properties and with family labor. It is difficult to determine precisely when it began, or the origin of the seeds used in the first plantations (Neitzke, 2012). For many decades, the crop was mainly used to produce dried pepper flakes, known as Calabrian pepper.

**Figure 9:**
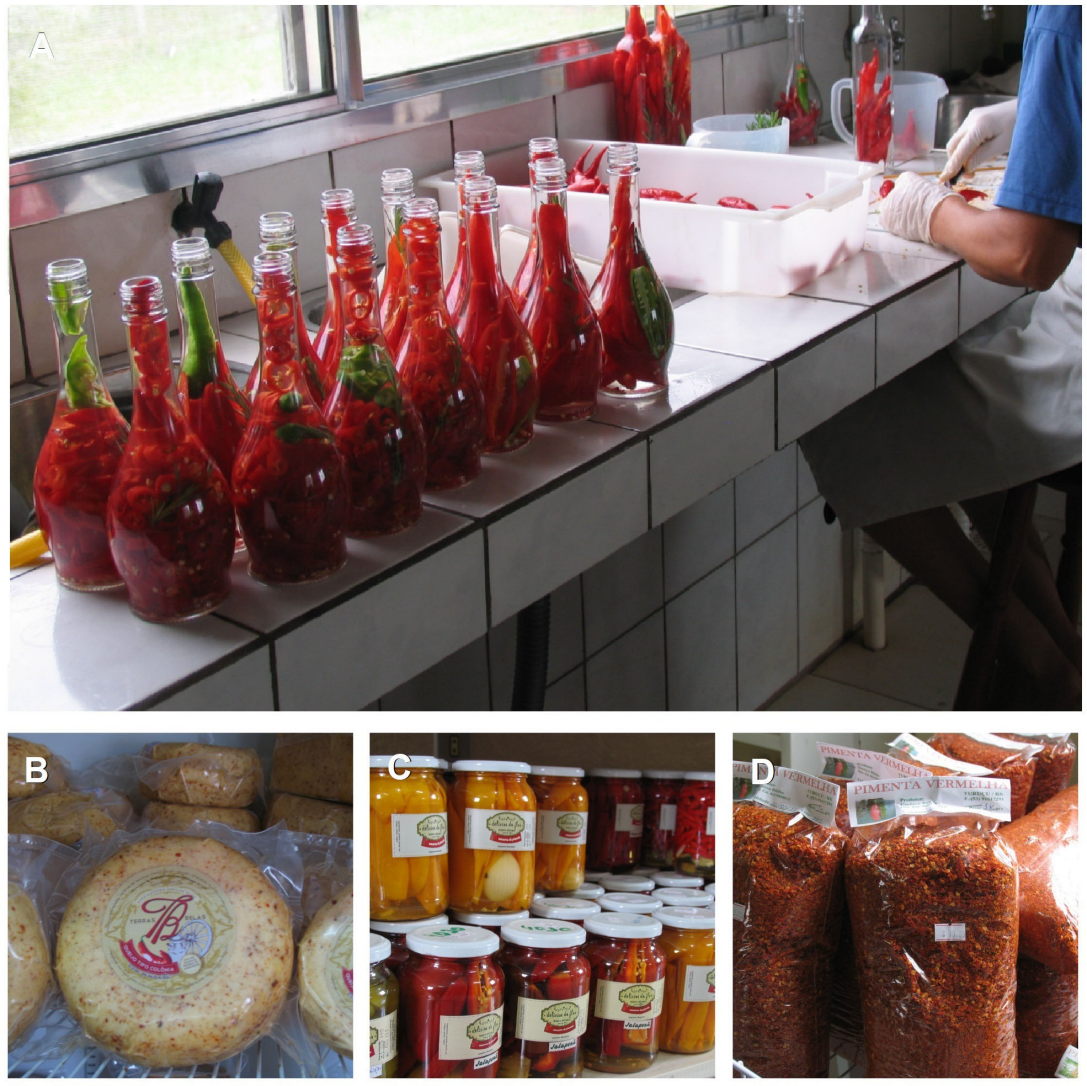
**(A)** Family agro-industry for processing *Capsicum* peppers; Products: **(B)** cheese with pepper; **(C)** pickled peppers; **(D)** dehydrated pepper flakes. Turuçu, Rio Grande do Sul, Brazil. (Pictures: Juliana Castelo Branco Villela).

In the early years of the 21st century, there was a major change in the pepper growing scenario in ‘Turuçu’. Many farmers abandoned the crop due to anthracnose (*Colletotrichum* sp.), a disease which is difficult to control. However, to remain in the pepper market, some producers set up family agro-industries and began to diversify the crop with the support of several public education and research institutions (Embrapa; Empresa de Assistência Técnica e Extensão Rural - Emater; Universidade Federal de Pelotas; and the Instituto Federal de Educação, Ciência e Tecnologia Sul-riograndense, Rio Grande do Sul). Before, they cultivated landraces of only one species (*C. baccatum*) and, from 2004, they started to cultivate landraces of more than three species (*C. frutescens, C. annuum* and *C. chinense*), with fruits of different sizes, shapes, colors, and pungency. As a result, more than 100 different pepper-based products are produced, such as pepper chocolates, various types of sauces, sweets, preserves, dehydrated peppers, among many others (**Figure 9**). While on the one hand there has been a reduction in the number of properties that cultivate peppers, on the other hand, there has been an increase in the genetic diversity of cultivated *Capsicum* and an increase in the diversity of marketed products. A large part of these products is marketed by the ‘Cooperativa Cooperturuçu’ in ‘Casa da Pimenta’, an establishment located in ‘Turuçu’. In this place, the cooperative farmers commercialize natural peppers, dried peppers and agroindustrial products. The ‘Casa da Pimenta’ is an excellent opportunity to sell and disseminate the products. The buyers come from different regions of Brazil, Argentina, and Uruguay. Another part of the products is marketed throughout Brazil (Villela et al., 2018).

**Figure 10:**
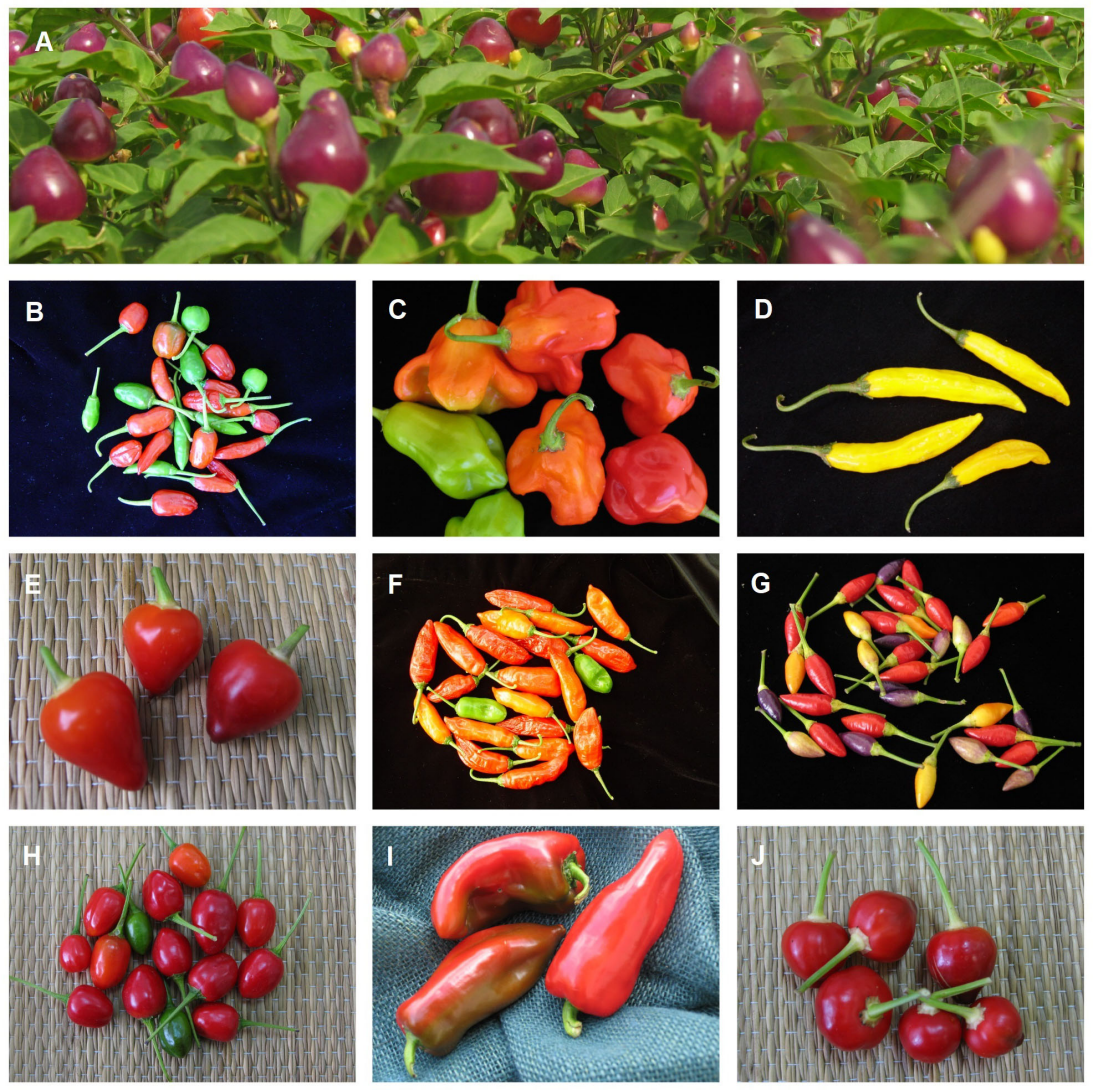
Landraces of *Capsicum annuum* **(A, B, E, G-J)**, *Capsicum baccatum* **(C, D)** and *Capsicum frutescens* **(F)** cultivated in the Brazilian Pampa biome. (Pictures: Rosa Lía Barbieri and Juliana Castelo Branco Villela).

According to the data available in the System ‘Alelo Vegetal’ (Embrapa recursos Genéticos e Biotecnologia, 2023), a total of 92 landraces of *Capsicum annuum, Capsicum baccatum, Capsicum chinense, Capsicum frutescens* cultivated by farmers in the Brazilian Pampas were collected from 2002 to 2019 for conservation in the *Capsicum* Genebank of Embrapa Clima Temperado (in Pelotas, Rio Grande do Sul). The characterisation and evaluation of pepper accessions from this genebank led to five doctoral theses (Neitzke, 2012; Vasconcelos, 2016; Padilha, 2017; Aranha, 2020; Cruz, 2022) and three master’s dissertations (Neitzke, 2008; Vasconcelos, 2012; Padilha, 2014). In general, these works have shown the wide genetic diversity in *Capsicum* landraces, with significant morphological variability, especially for fruit color, shape, and size (**Figure 10**), as well as different levels of pungency, total phenolic compounds, *in vitro* antioxidant activity, total anthocyanins and total carotenoids. Vasconcelos (2016) observed variation in the post-harvest quality of ripe fruit. The study of the relationship between pepper fruit compounds in response to the pathogen *Colletotrichum* sp. generated information on the process of resistance and susceptibility (Padilha, 2017). Molecular characterization of *C. baccatum* landraces was performed by Villela et al. (2014).

**Figure 11:**
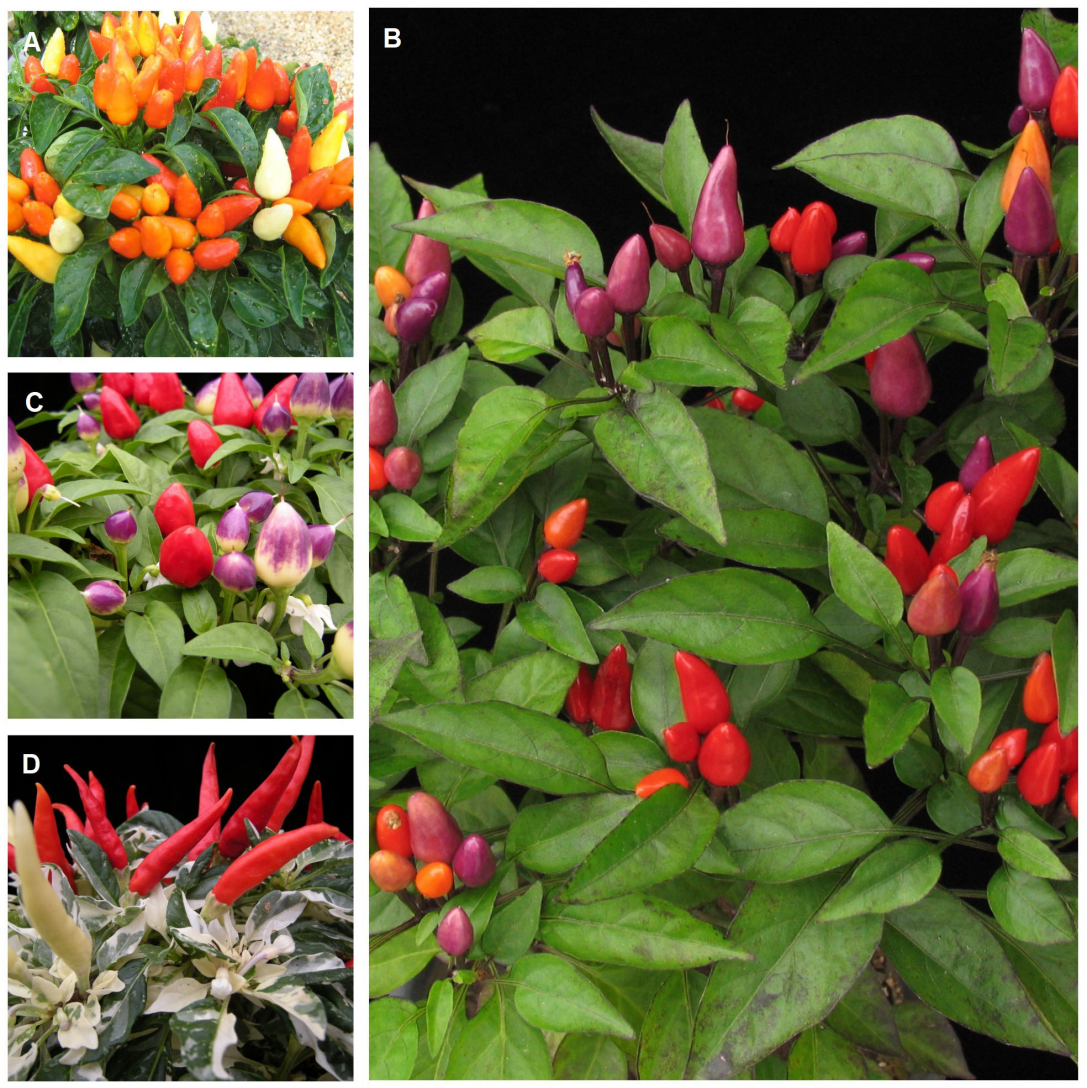
Landraces of ornamental pepper (*Capsicum annuum* - **A–D**) cultivated in the Brazilian Pampa biome. (Pictures: Rosa Lía Barbieri, Raquel Silviana Neitzke and Henrique Kuhn Massot Padilha).

Neitzke (2012) focused on the evaluation of landraces of peppers for ornamental use (**Figure 11**). Her study verified that the ornamental use is certainly due to their aesthetic value (plant architecture; quantity, shape, and position of fruits; coloring, shape and density of leaves and fruits), as well as how easily they may be grown and how long they maintain their ornamental appearance in a pot, including the durability of fruits and leaves, as well as continuous fruit production. Small-sized landraces are especially desirable for cultivation in pots and flowerbeds. Both small-sized and medium to tall landraces can be used in landscaping (Neitzke et al., 2010). When evaluating the acceptance of ornamental pepper landraces grown in pots by 200 potential consumers, with a predominance of women (74.5%), a greater preference for plants with contrasting color fruits in relation to the foliage was verified.

#### 3.3.5 A synthesis of the four crop case-studies

**Figure 12:**
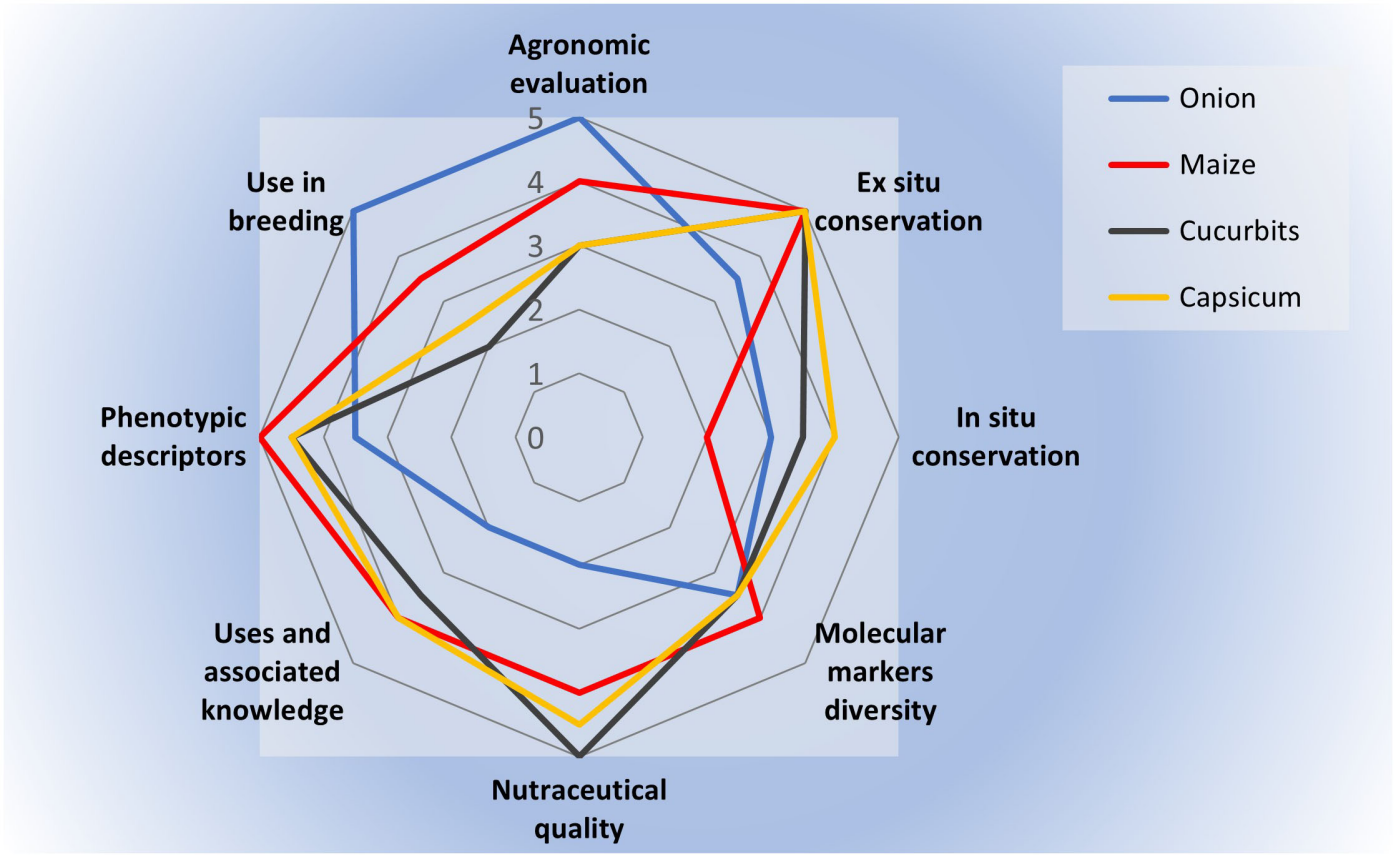
A synthesis of the research progress across several goals and aspects in the four crops presented as case studies, from 1 (little progress) to 5 (high progress).

In **Figure 12** we summarize the strength and weakness of landraces research in the four cases previously presented. In all cases, *ex situ* collections were established, though for onion the collection has suffered erosion and has no background. Phenotypic characterization, as well as molecular and nutritional is also variable, though studies have been made. A general constrain is the lack of physiological studies describing specific adaptations. Agronomic evaluation and landraces use in formal breeding is advanced for onion and maize. Ethnobotanical research is scant, though recent advances were obtained for maize landraces. *Capsicum* landraces stand out in the promotion of the use of diversity. *In situ* conservation and complementarity with *ex situ* conservation is a challenge in all cases.

#### 3.3.6 Landraces of specific minor crops

One example of traditional crops in family farming systems is grass pea (*Lathyrus sativus*), locally called *‘chícharo’* in Uruguay. It is a NUS crop (Hernández et al., 2019) which has been revived and promoted for its use (Galván et al., 2014). Grass pea is a winter-growing legume integrated as staple food crop in rural areas for decades, with both the green and dry pulses being utilized. The production of flour is another important use in Uruguay. Grass pea landraces were introduced by immigrants from Europe, likely from Spain and Italy (Galván et al., 2005). Four grass pea landraces were evaluated for grain yield and agronomic performance in different environments (Galván et al., 2014). Differences in time to flowering onset and duration, plant height, plant weight, and grain yield were recorded. An accession flowering earlier had a smaller plant size and determinate growth, while a longer cycle and flowering time showed higher yields across environments, pointing to a rich genetic diversity among grass pea landraces.

Another interesting case is the *‘feijao de vagem*’, pod beans (*Phaseolus vulgaris*), a variety of the common bean. The pods possess a low fiber content, rendering them more tender and suitable for consumption when cooked or boiled. There are landraces exhibiting either determinate or indeterminate growth habits, flattened or cylindrical pods with yellow, green, or purple color. Within the existing bean collection at Embrapa Clima Temperado, there are ten accessions of pod beans originating from the Pampa and Atlantic Forest biomes, with seven of them conserved by female guardians and three by male seed guardians. These landraces display different combinations of size, color, luster, and shape in their seeds.

In Uruguay, peanuts (*Arachis hypogaea*) have traditionally been cultivated using landraces. A survey and collection of 285 landraces recorded variations in fruit color, number of kernels per fruit, pod size, and shape (Millot and Cairús, 1985). Since the 1980s, peanut cultivation has decreased due to rural emigration (Pereira, 1995). Currently, peanuts are cultivated only by family farmers using landraces in a traditional manner (Courdin et al., 2010; Barreto Mendizabal, 2014).

## 4 Strategies towards an enhanced identification, conservation, and valorization of landraces

### 4.1 Which landraces, where, and whose hands own landraces?

Based on the information regarding territories where farmers conserve landraces, the crop case studies, and *ex situ* collections, it can be affirmed that horticultural landraces are widely present in the family farming systems of the Pampa biome, both for self-consumption and commercial purposes. The results presented are consistent with Jarvis et al. (2011), who argue that farmers still maintain significant genetic diversity for a set of globally important crops.

There is a high number of cultivated species in which production and consumption are based on landraces. Landraces of maize, sweet potatoes, beans, various species of *Cucurbita* and *Capsicum*, onions, and tomatoes prevail. The diversity of these food crops, including leafy vegetables (*Beta vulgaris* var. *cycla, Lactuca sativa, Eruca sativa, Brassica* spp., among others), is highlighted by farmers in this analysis. In most family farms, landraces of aromatic crops (*Ocimum basilicum, Coriandrum sativum, Anethum graveolens, Salvia* spp., among others) are also conserved. The diversity of landraces conserved *in situ* is reflected not only in phenotypic and genetic diversity, but also in the diversity of uses and names by which farmers call them (Costa et al., 2010; Burgueño et al., 2015; Mello et al., 2017; Pereira, 2017; Cuadro, 2022).

Although the surveys conducted and described here represent progress in knowledge, they also indicate the need to generate an inventory to establish the status of *in situ* conservation in the Pampa biome, as the surveyed sites are still very few and, in some cases, only a few crops’ landraces were surveyed. This inventory would allow the identification of priority territories for *in situ* conservation (hotspots or microcenters of diversity) and facilitate the necessary complementation between *in situ* and *ex situ* conservation. Landraces are dynamic populations (Brush, 1991; van Heerwaarden et al., 2012), so inventories should also be dynamic through systematic monitoring of genetic erosion processes (Brush, 1999; Brown, 2008). The systematic survey would enable the rescue of threatened landraces and NUS for *ex situ* conservation and for potential multiplication and distribution to other farmers. This strategic proposal could be implemented by creating observatories in different locations with the participation of farmers.

The inventories are set out in Article 5 of the ITPGRFA (FAO, 2001) and in the Second Global Plan of Action for Plant Genetic Resources for Food and Agriculture (FAO, 2011) as an essential tool for conservation. This priority activity is based on the historical lack of integration between research, monitoring, and conservation efforts, which ultimately impacts the effectiveness of conservation (Maxted et al., 2009; Khoury et al., 2022). The relevance of these inventories lies in the absence of information regarding the crops and locations where landraces are utilized in production. Without this knowledge, it becomes exceedingly challenging to develop effective conservation and promotion measures (Raggi et al., 2022).

The inventory of landraces and associated knowledge allows the definition of territories to focus on *in situ* conservation. *In situ* conservation is understood as a dynamic conservation integrated with its biophysical, socioeconomic, and cultural environment, where evolutionary and coevolutionary processes, as well as selection and diversification processes in agroecosystems, continue (Maxted et al., 1997; Rivas et al., 2010). The efforts towards *in situ* – *on farm* conservation should focus on territories with the highest diversity of landraces in different crops, although this does not preclude directing efforts towards specific crops.

### 4.2 *In situ – on farm* conservation

Microcenters of diversity or genetic reservoirs are part of biocultural landscapes (Hong, 2014), which require an integrated approach to biological and cultural diversity for their conservation (Pretty et al., 2009). Community-based *in situ* conservation tools, where farmers play a central role, include agrobiodiversity zoning and designating parks or areas as protected landscapes.

To conserve agrobiodiversity, it is crucial to value farmers and their culture as the main contributors to the generation and maintenance of landraces, which are a legacy for present and future generations (Cassino et al., 2019). *In situ* – *on farm* conservation is carried out with active farmer participation, and a balance between ideal conservation practices and farmers’ economic and cultural demands is required (Rivas, 2001; Jarvis et al., 2011; Bellon et al., 2015). This conservation of plant genetic resources is linked to the conservation of the overall natural resources (Qualset et al., 1997), and the objective is not solely to safeguard genetic materials but to benefit farmers and ensure the continuity of agroecosystems (Jana, 1993). An effective conservation needs to be actively planned, designed, monitored, and valued (Maxted et al., 1997).

Maintaining *on-farm* diversity should not come at the expense of farmers, who, in certain situations, may decide to abandon their activities or replace landraces with modern cultivars. Negative incentives, such as granting credit based on the use of improved cultivars or technological changes driven by both public and private sectors can lead to such situations (Qualset et al., 1997). The conservation of agrobiodiversity is a public benefit, requiring positive incentives such as the implementation of *in situ* conservation projects (Bellon et al., 2015). Consequently, in recent decades, national and international NGOs, farmers organizations, foundations, universities, and research organizations have implemented *in situ* conservation projects worldwide.

The cultivation of landraces is mostly carried out on relatively small properties by families which are deeply rooted in the territory. To ensure the continuity of these productive systems, which are heavily threatened by the lack of generational turnover, incentives are needed to encourage young people to participate in productive activities and prevent them from migrating to cities in search of other opportunities. This issue should be addressed by public policies aimed at conserving and developing innovative alternatives based on the valorization of landraces that ensure that their production is a profitable business. Some authors argue that the only way to ensure long-term *in situ* conservation is for new farmers to adopt these landraces for cultivation (Maxted et al., 2009; Casañas et al., 2017; Raggi et al., 2021). In this sense, focusing on young people and newcomers to rural areas is crucial to maintain and use landraces sustainably.

### 4.3 Rediscovery and valorization of landraces as a sustainability key

Under the premise that facilitating and promoting the use of landraces is the most effective approach to mitigate their genetic erosion, the concept of conservation through their utilization in value chains is reinforced (Raggi et al., 2021).

Community-based conservation tools are diverse. Jarvis et al. (2011) identified 59 different types of interventions, whose applicability and success depend on farmers, farming communities, and local institutions. On the other hand, Bellon et al. (2015) proposed a theoretical framework for analyzing the degree of success of different types of interventions, considering both private and public benefits. Projects that provide farmers with technologies, capacity building, and organizational structures for access, management, and marketing would be the most suitable and comprehensive approach.

The factors identified by Raggi et al. (2021) as the most relevant to add value and increase accessibility to landraces include the multiplication of seeds by organizations or institutions, thus complementing the multiplication carried out by farmers with additional efforts, perspectives, and resources. The applicability of this instrument in the Pampa biome is supported by a situation in which many landraces are held by only one or a few farmers who, due to their age or economic situation, may abandon productive activities at any time. Networks and institutions in this regard should be responsible for ensuring the existence of seed volumes for distribution among interested farmers, as well as for *ex situ* conservation, maintaining the local adaptation of these landraces and the quality of the obtained seeds. Technical support and training for farmers in seedbed management and seed conservation should also be considered.

‘Community seed banks’ or ‘seed houses’ play an important role in safeguarding landrace seeds (Silva, 2013; Santos et al., 2015; Vernooy et al., 2015; Pádua et al., 2022). In the Pampa region, some of these banks have been established, and managed by local groups of farmers who are members of different networks or organizations. To ensure their continuity and stability, these banks face significant challenges in terms of the social and governmental recognition of their seed guardians, as well as regarding the allocation of a minimum budget to operate.

A second factor related to accessibility and added value is the integration of landraces into sales and marketing channels and not exclusively for self-consumption (Raggi et al., 2021), as landraces with limited use are unlikely to gain recognition and generate interest in their conservation. Hence, real utilization becomes a key factor. While surveys and catalogs can help to visualize landraces diversity and contribute to their valorization, there are very few landraces in the Pampa biome that are commercially recognized. Among these, *Phaseolus vulgaris* ‘Liebrero’ or ‘Azufrito’, *Phaseolus lunatus* ‘Poroto sopa’, *Vigna unguiculata* ‘Tape’, *Cucurbita pepo* ‘Zapallo criollo’, as well as cultivars derived from landraces such as *Cucurbita maxima* ‘BRS Tortéi’ and ‘BRS Linda’, and *Allium cepa* ‘Pantanoso del Sauce’ stand out, along with others with potential such as *Lathyrus sativus* landraces, highly appreciated for their taste. Integrating them into supply chains could allow for the inclusion of geographical indications and trademarks, like the case of the Italian landrace ‘Aglione della Val di Chiana’ of *Allium ampeloprasum*, or the Finnish landrace of ‘Puikula’ potatoes, among others (Raggi et al., 2021). Significant investment is required to replicate these experiences and enhance landraces value.

In addition, Raggi et al. (2021) associate conservation with promotion, reaffirming the concept that one of the best ways to conserve landraces is through their utilization. Creating alliances with consumer associations is a strategy to link effective commitment to landraces conservation with healthy nutrition, contributing to the economic viability of their conservation. To progress in this valorization strategy, research is required on genetic diversity and phenotypic, nutritional, and organoleptic characterization. These studies would enable distinctive identification and the development of technological packages for specific landraces (Azeez et al., 2018). The proposal is therefore to conduct joint research with farmers on the best agroecological practices to ensure quality production of food and seeds.

The policy of combining the objectives of landrace conservation and sustainable use with the development of agroecology is a highly encouraging and natural path, especially considering that a significant proportion of farmers maintaining landraces engage in agroecological production. The National Plan for Agroecology and Organic Production (2012) in Brazil, and the National Plan for the promotion of production based on agroecological principles (2018) in Uruguay, prioritize the conservation of plant genetic resources/socio biodiversity and propose various programs and projects, such as promoting government purchases (by hospitals, prisons, children’s shelters, etc.), expanding and promoting marketing in street markets and specialized stores, giving incentives to add value, strengthening participatory guarantee systems and participatory plant breeding.

The development of high-quality artisanal products and specialized stores has shown to be a successful alternative. An example is the case of peppers as presented in this review, an initiative by ‘Cooperturuçu’ which, with initial support, was able to develop a set of attractive products with an appropriate marketing channel, ensuring the conservation of *Capsicum* landraces. Seed festivals and fairs for native and landrace seeds held in the region serve as instances for farmers to gather and exchange valued seeds. These activities could be expanded and enhanced to increase social visibility for the conservation of agrobiodiversity in the context of agroecological production and healthy nutrition. Showcasing traditional dishes prepared by farmers, innovative dishes created by chefs, manufactured products, attractive handicrafts, and cultural products would be alternative approaches to be more implemented and in more locations.

Obtaining designations of origin or provenance is proposed as another mechanism for valorizing landraces. However, in our countries there is little experience in this area, so it does not seem to be a viable option in the short term (Luthy, 2015). It is still recommended to integrate and train intellectual property experts with experts in agrobiodiversity to advance the generation of *ad hoc* mechanisms that are suitable for our farmers. Working on branding is more feasible than developing designations of origin, both in collective and participatory certification terms (Luthy, 2015). This is an issue not yet addressed by farmers, with the associated difficulty of costs related to certification systems.

Another valorization tool is participatory plant breeding, which is still in its early stages for landraces in our countries (Pádua et al., 2022). However, genetic improvement carried out in crops such as onion and maize using germplasm from landraces has yielded positive results, particularly because these crops and objectives have not been prioritized in the more predominant formal breeding programs. Casañas et al. (2017) discuss the relationship between formal systems (institutional and corporate) and community-based maintenance systems of landraces. The coexistence of diverse systems in the use and management of crop genetic resources is widely recognized (Lammerts van Bueren et al., 2018). This coexistence creates connections, leading Casañas et al. (2017) to propose expanding the concept of landrace to include formal genetic improvements based on landraces, even with the incorporation of specific genes through backcrossing. The traditional maintenance of landraces by family farmers preserves genetic richness and resilience, and contributes to integrated plant breeding systems, addressing global challenges like climate change and food security (Lammerts van Bueren et al., 2018).

Education and training on issues related to the valuation, conservation and sustainable use of plant genetic resources are key aspects of any strategy that seeks to enhance the value of landraces (Bellenda et al., 2018). Formal education programs at the primary and secondary levels as well as non-formal education for the general public should highlight conservation of agrobiodiversity as a goal, and landraces diversity in particular, pointing to their contribution to food security, food sovereignty, and nutraceutical properties. University education both in degree and postgraduate levels should increase the attention to agroecology and landrace diversity, which implies a range of careers due to inter and transdisciplinary challenges. Even when progress is observed in scientific literature, courses and specializations are important to enlarge critical mass. Researchers and professionals trained in *in situ* conservation, management of genebanks and community banks, characterization (phenotypic, nutritional, genetic and ethnobotanical), plant breeding and participatory landraces breeding, development of local trades and appellations of origin, product and gastronomic innovations, agroecological management, among other aspects, including legal ones, are needed. Exchange of knowledge with and among farmers and capacity building is another strategy to enrich, using the classical divulgation texts and videos up to the internet social media (Hasan and Abdullah, 2015; Volk et al., 2019; Comité Nacional de Recursos Fitogenéticos, 2021; Abreu et al., 2022).

### 4.4 A growing friendship between *in situ* and *ex situ* conservation of landraces

Based on the state of *ex situ* collections of vegetable landraces, implementing a strategic goal of expanding territorial coverage through new collection is proposed (Comité Nacional de Recursos Fitogenéticos, 2021; Abreu et al., 2022). The studies presented here on the distribution of genetic diversity in some crop landraces along with expert knowledge, would help identify gaps and duplicates to plan new collections (Ramírez-Villegas et al., 2020). Additionally, revisiting the sites where the accessions in genebanks originate from is proposed to assess genetic erosion processes and obtain new samples to enhance the understanding of these population’s dynamics.

Characterization and agronomic evaluation of collections, along with seed regeneration and multiplication activities, are essential for valorizing landraces and providing seeds for farmers in need, as well as fostering new productive ventures based on landraces. Increasing the connection between genebanks and farmers is a short-term goal, requiring the establishment of contracts to uphold mutual commitments of bidirectional seed exchanges between banks and farmers, ensuring volume and seed quality.

Repatriation of collections preserved in other national or international banks, but not present in our countries for various reasons, also deserves attention. Examples include the maize collection from Uruguay kept at the ‘INTA Pergamino’ genebank (Argentina) (Jaurena et al., 1999) and peanut accessions from the ‘USDA’ collection (United States) (Naya et al., 2022). In this regard, further development of the ITPGRFA’s Multilateral System of Access and Benefit Sharing is warranted, although both Brazil and Uruguay are already participants.

### 4.5 Regulatory frameworks and concluding remarks

The relevance of agrobiodiversity, particularly landraces, is recognized in international instruments such as the Convention on Biological Diversity (CBD) (United Nations, 1992), the International Treaty on Plant Genetic Resources for Food and Agriculture (ITPGRFA) (FAO, 2001), the Second Global Plan of Action for Plant Genetic Resources for Food and Agriculture (GPA) (FAO, 2011), and the Voluntary Guidelines for the Conservation and Sustainable Use of Farmers’ Varieties/Landraces (FAO, 2020). In our countries, which are signatories to the CBD and the ITPGRFA, the challenges related to landraces are still timidly present in the public sector, depending on the actions of family farmers’ organizations, NGOs and academia.

Based on the analysis of the main tools to support the valorization of landraces, it is relevant to establish the most suitable governance framework that ensures the participation of farmers, academia, and public institutions, and enables effective progress in an action plan for the conservation, sustainable use, and effective implementation of farmers’ rights (FAO, 2011; FAO, 2021).

Regulations in Uruguay and Brazil allow the free exchange of landrace seeds, but show differences in case of selling the seeds, as in Uruguay a previous registration of the landrace is required (www.inase.uy), whereas in Brazil selling landrace seeds among farmers or farmers organizations is fully permitted. While these regulations comply with Article 9.3 of the International Treaty on Plant Genetic Resources for Food and Agriculture (ITPGRFA), further measures are needed as proposed by the ‘Inventory of national measures, best practices and lessons learned from the realization of Farmers’ Rights, as set out in Article 9 of the International Treaty’ (www.fao.org/plant-treaty/tools/toolbox-for-sustainable-use/details/en/c/1473076/).

This article highlights the status of conservation, use, and valorization of vegetable landraces in the Pampa biome, addressing the main socioeconomic and environmental challenges as a starting point to strengthen ongoing lines of action and generate new proposals aligned with the 2030 Agenda for Sustainable Development (United Nations, 2015) and the Aichi Biodiversity Targets (United Nations, 2011), especially Target 13, which sets out that “the genetic diversity of cultivated plants and farmed and domesticated animals and of wild relatives, including other socio-economically as well as culturally valuable species, is maintained, and strategies have been developed and implemented for minimizing genetic erosion and safeguarding genetic diversity”. These new proposals require joint and coordinated efforts among governmental actions, academia, civil society organizations, and, particularly, the involvement of farmers.

## Author contributions

MR - study conception and design, data collection, analysis and interpretation of results, draft manuscript preparation and final edition. RV - data collection, analysis and interpretation of results, draft manuscript preparation. RN - data collection. DP - data collection. NA - data collection. IF - data collection. GG - data collection, analysis and interpretation of results, draft manuscript preparation and final edition RB - study conception and design, data collection, analysis and interpretation of results, draft manuscript preparation and final edition. All authors contributed to the article and approved the submitted version.
